# Renal remodeling by CXCL10-CXCR3 axis-recruited mesenchymal stem cells and subsequent IL4I1 secretion in lupus nephritis

**DOI:** 10.1038/s41392-024-02018-5

**Published:** 2024-11-18

**Authors:** Qixiang Zhang, Yunlong Shan, Luping Shen, Qi Ni, Dandan Wang, Xin Wen, Huanke Xu, Xiaoyan Liu, Zhu Zeng, Jingwen Yang, Yukai Wang, Jiali Liu, Yueyan Su, Ning Wei, Jing Wang, Lingyun Sun, Guangji Wang, Fang Zhou

**Affiliations:** 1 Key Laboratory of Drug Metabolism and Pharmacokinetics, Haihe Laboratory of Cell Ecosystem, State Key Laboratory of Natural Medicines, China Pharmaceutical University, Nanjing, China; 2 Department of Pharmacy, Zhongshan Hospital, Fudan University, Shanghai, China; 3 Department of Rheumatology and Immunology, The Affiliated Drum Tower Hospital of Nanjing University Medical School, Nanjing, China; 4 Jiangsu Renocell Biotech Co., Ltd., Nanjing, China

**Keywords:** Mesenchymal stem cells, Immunological disorders, Kidney diseases

## Abstract

Human umbilical cord mesenchymal stem cells (hUC-MSCs) have shown potential as a therapeutic option for lupus nephritis (LN), particularly in patients refractory to conventional treatments. Despite extensive translational research on MSCs, the precise mechanisms by which MSCs migrate to the kidney and restore renal function remain incompletely understood. Here, we aim to clarify the spatiotemporal characteristics of hUC-MSC migration into LN kidneys and their interactions with host cells in microenvironment. This study elucidates that the migration of hUC-MSCs to the LN kidney is driven by elevated levels of CXCL10, predominantly produced by glomerular vascular endothelial cells through the IFN-γ/IRF1-KPNA4 pathway. Interestingly, the blockade of CXCL10-CXCR3 axis impedes the migration of hUC-MSCs to LN kidney and negatively impacts therapeutic outcomes. Single cell-RNA sequencing analysis underscores the importance of this axis in mediating the regulatory effects of hUC-MSCs on the renal immune environment. Furthermore, hUC-MSCs have been observed to induce and secrete interleukin 4 inducible gene 1 (IL4I1) in response to the microenvironment of LN kidney, thereby suppressing Th1 cells. Genetically ablating IL4I1 in hUC-MSCs abolishes their therapeutic effects and prevents the inhibition of CXCR3^+^ Th1 cell infiltration into LN kidneys. This study provides valuable insights into the significant involvement of CXCL10-CXCR3 axis in hUC-MSC migration to the LN kidneys and the subsequent remodeling of renal immune microenvironment. Regulating the CXCL10-CXCR3 axis and IL4I1 secretion may be developed as a novel therapeutic strategy to improve treatment outcomes of LN.

## Introduction

Lupus nephritis (LN) is a common severe complication of systemic lupus erythematosus (SLE) and is a high-risk factor for mortality.^1–3^ In all, 60–80% of SLE patients develop renal disorders of differing severity during the course of their illness.^4^ More than 50% of treated patients with LN have been reported to be refractory to standard immunosuppressive therapy,^5^ which means worse long-term renal outcomes and a lower survival rate. Even patients who respond to standard treatment regimens often experience severe drug side effects or toxicity. Thus, there is still an urgent need for novel therapy.

Mesenchymal stem cells (MSCs), including umbilical cord-derived MSCs (UC-MSCs), bone marrow-derived MSCs (BM-MSCs), and so on, are undergoing intensive translational research for some critical or refractory diseases such as graft versus host disease (GvHD), acute respiratory distress syndrome (ARDS) and SLE. MSCs have also emerged as a promising therapy for LN because they can ameliorate disease activity and renal functions besides their potent immunosuppressive effects in both preclinical and clinical studies.^6–9^ A small number of reports about inconsistent therapeutic effects may be partly attributed to the varied resources of MSCs, non-uniform preparation processes, various disease severity, or different clinic therapeutic regimes.^10,11^ The efficacy of MSCs in LN treatment remains to be confirmed. Furthermore, how can MSCs improve renal function is still unclear.^12^ Previous studies have primarily examined the immune regulatory effects of MSCs on isolated immune cells or peripheral blood mononuclear cells (PBMC).^13^ Consequently, it is crucial to investigate the mechanisms through which MSCs exert their reparative effects on the constituent cells in the kidney and improve renal function.

To fully exploit the therapeutic capabilities of MSCs and effectively apply them as advanced medicinal products in clinical settings, it is imperative to possess a comprehensive understanding of MSC distribution, retention, and their ultimate fate within the host organism and the damaged tissues.^14^ Clinical applications of MSCs are reliant on their homing ability and successful migration to the sites of inflammation and the injured tissue after administration.^15,16^ Unfortunately, MSC homing seems inefficient,^17^ and the precise regulatory mechanisms in the response of MSCs to special injury signals in different diseases are still unclear. These may be a major bottleneck in realizing the full therapeutic potential of MSC-based therapies.

MSCs can express a wide range of chemokine receptors and the soluble ligands of these receptor chemokines may play a key role in the homing of MSCs.^18^ Among these chemokines and the corresponding receptors, the CXCL12-CXCR4 axis was most studied in MSC therapy for various diseases.^19–22^ Some studies found that CXCL12-CXCR4/CXCR7 mediated MSC homing to the ischemia/reperfusion-injured kidney.^23–25^ However, some reports showed that MSCs did not express CXCR4, suggesting other receptors and chemokines were involved.^26,27^ Consequently, the expression patterns of these receptors on MSCs may vary depending on the different inflammatory statuses of individuals who undergo engraftment, thereby influencing the migration of MSCs toward specific tissues. Following the migration of MSCs to LN kidneys, their interactions with host cells in microenvironment remain incompletely understood, which may also have a significant impact on the outcome of MSC treatment.

T cells comprise the majority of kidney-infiltrating immune cells in LN with an exhausted phenotype.^28^ Th1 cells, which are present in the inflamed kidneys and contribute to the progression of nephritis, are of particular interest.^29^ In addition to Th1 cells, the activation of Th17 cells and functional deficiencies in Treg cells are also found to be related to glomerular damage in LN.^30–32^ Recent studies have demonstrated the involvement of CXCL10-mediated immune cell recruitment in the pathogenesis of certain renal diseases.^29^ Specifically, the CXCL10-CXCR3 axis has been identified as a critical mediator of renal CXCR3^+^ T cell infiltration in LN, thereby contributing to disease progression.^33^ However, the factors driving hUC-MSC homing to the LN kidneys remain unclear. Furthermore, a comprehensive understanding of the interactions between MSCs and the immune microenvironment in LN kidneys is essential.

To address the aforementioned concerns regarding the application of hUC-MSCs in LN therapy, we initially investigated whether the spatiotemporal distribution patterns of hUC-MSCs after administration were consistent with the dynamic improvement of renal function. Next, the underlying mechanisms by which hUC-MSCs migrate to the LN kidneys were explored from the spatiotemporal characteristics of MSC distribution and their interactions with host cells in LN kidneys. Then, the roles of recruited hUC-MSCs in restoring renal immune homeostasis were further investigated.

## Results

### Increased recruitment of hUC-MSCs to kidneys alleviates renal injury of lupus nephritis

Migration of MSCs to the renal lesion site and the retention time in vivo are important factors in their therapeutic effect. When MRL/lpr mice developed symptoms of LN, the mice received vein injections of hUC-MSCs. After administration, the biodistribution of MSCs in different organs at a series of time points was quantitatively determined by a validated Q-PCR method based on previous studies.^34–36^ And pharmacodynamic indicators related to LN were continuously monitored for 1 month.

**Fig. 1:**
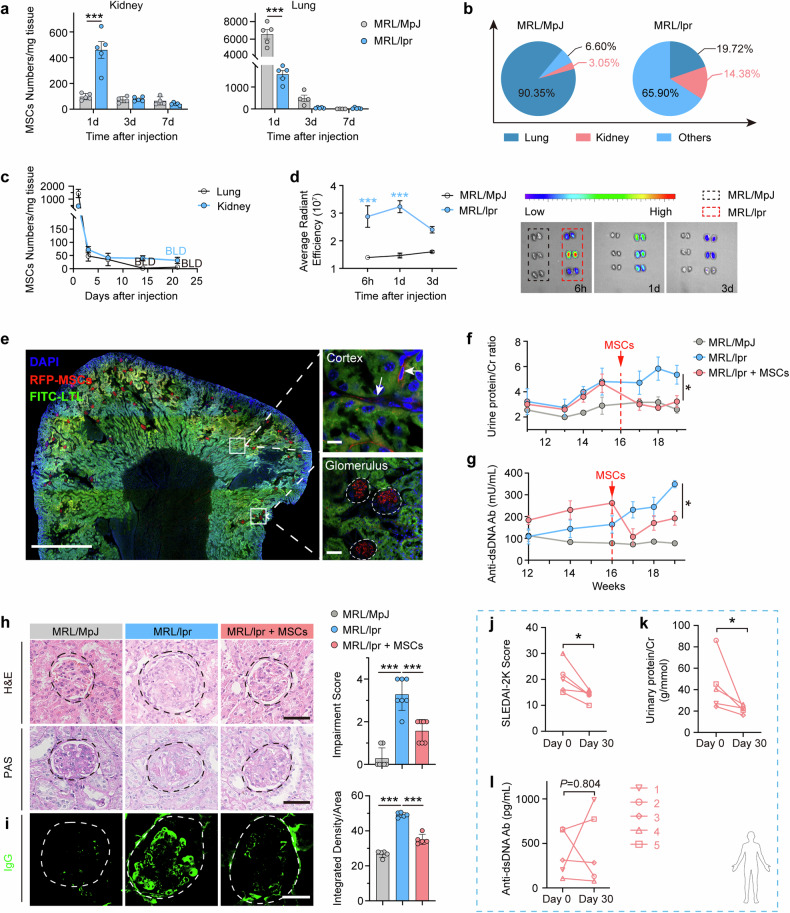
Increased recruitment of hUC-MSCs to kidney in MRL/lpr mice alleviates renal of lupus nephritis. **a** The quantities of hUC-MSCs were assessed by Q-PCR in the lungs and kidneys of MRL/lpr and MRL/MpJ mice at 1 day, 3 days, 7 days after intravenous transplantation of hUC-MSCs (*n* = 4–5). **b** Proportional distribution of hUC-MSCs in the lungs, kidneys, and other tissues at 1 day after intravenous transplantation. **c** The line plots illustrating the quantity of MRL/lpr mice at 1 day, 3 days, 7 days, 14 days and 21 days after intravenous injection (*n* = 4–5). BLD means below the low detection. **d** Fluorescence intensity of RFP-hUC-MSCs (the hUC-MSCs expressing RFP fluorescent protein) in kidneys is determined by IVIS Imaging System (*n* = 3). **e** Representative images of RFP-hUC-MSCs distribution in kidney sections from MRL/lpr mice. FITC-LTL is administered intravenously. Scale bar: full scan of kidney section (1000 µm), Glomerulus (50 µm), Cortex (10 µm). **f** Dynamic curves of urine protein/creatinine levels in MRL/MpJ and MRL/lpr mice treated with or without hUC-MSCs (*n* = 7). **g** Dynamic curves of serum anti-dsDNA antibody levels in MRL/MpJ and MRL/lpr mice treated with or without hUC-MSCs (*n* = 7). **h** Glomerular pathological sections of MRL/lpr and MRL/MpJ mice treated with or without hUC-MSCs (*n* = 5). Scale bar: 50 µm. **i** Deposition of immune complexes IgG in glomerulus of MRL/lpr mice treated with or without hUC-MSCs (*n* = 5). Scale bar: 30 µm. **j**–**l** SLEDAI-2K Score (Systemic Lupus Erythematosus Disease Activity Score) (**j**), urine protein/creatinine levels (**k**), anti-dsDNA antibodies levels (**l**) in 5 lupus nephritis patients before and 30 days after treatment with hUC-MSCs. *P < 0.05, ***P < 0.001

On Day 1 after administration, there was a significant recruitment of MSCs to the kidneys of LN mice (4.72 times of the control group, Fig. 1a). The proportion of kidney distribution quantity to the total MSC dosage was increased from 3.05% in MRL/MpJ control mice to 14.38% in MRL/lpr LN mice (Fig. 1b). In addition, MSCs had a longer retention time (>14 days) in diseased kidneys than that in lungs which was under the low limit of detection on day14 (Fig. 1c). Similar results were obtained by IVIS Imaging of hUC-MSCs with stable expression of RFP fluorescent (Fig. 1d). MRL/lpr mice showed a significantly reduced fluorescence intensity in the lungs and a notably enhanced fluorescence intensity in the lymph nodes when compared to MRL/MpJ mice, while the fluorescence intensity in the heart, liver, and spleen was virtually undetectable (Supplementary Fig. 1a, b). As shown in Fig. 1e, RFP-MSCs (red) mainly distributed in the glomerulus in the sections of LN kidneys, while RFP fluorescent signals failed to be detected in the kidneys of MRL/MpJ mice (Fig. 1e and Supplementary Fig. 1c).

For evaluating the efficacy of MSC treatment, biochemical indicators in urine and serum were continuously monitored as well as the pathology study at the endpoint. It was found that hUC-MSCs treatment significantly decreased the levels of urine protein/creatinine and serum anti-dsDNA antibodies in LN mice, and the levels were consistently lower than those in MRL/lpr LN mice (Fig. 1f, g). Compared with control MRL/MpJ mice, MRL/lpr mice exhibited significant glomerulosclerosis and IgG disposition. These pathologic changes were effectively alleviated in MRL/lpr mice treated with hUC-MSCs (Fig. 1h, i), and the splenic index of mice was also significantly improved (Supplementary Fig. 1d). Moreover, MRL/lpr mice were also effectively relieved in skin damage after treatment with hUC-MSCs (Supplementary Fig. 1e). Additionally, five patients with a diagnosis of refractory LN were enrolled in hUC-MSC treatment. Prior to the hUC-MSC infusion, these patients discontinued immunosuppressive therapy and were transitioned to a regimen of low-dose glucocorticoids. These patients were closely monitored during the treatment (Table 1). After a period of thirty days following the administration of hUC-MSCs, reductions were observed in the SELDAI scores of five patients (No. 1–5), as well as in the levels of urine protein/creatinine and blood creatinine (Fig. 1j, k and Supplementary Fig. 1f). Furthermore, among the five patients undergoing hUC-MSCs therapy, three patients experienced a decrease in serum anti-dsDNA antibody and blood urea nitrogen (BUN) levels, and two patients experienced an increase in serum anti-dsDNA antibody (No. 1 and No. 5) and blood urea nitrogen (BUN) (No. 3 and No. 4) levels (Fig. 1l and Supplementary Fig. 1g).

**Table 1:** Clinical characteristics of LN patients

Terms	LN-1	LN-2	LN-3	LN-4	LN-5
Age/gender	32/F	34/F	31/F	22/F	37/F
Disease duration (month)	12	108	24	22	36
SLEDAI-2K Score	20	22	16	30	15
Clinical characteristics					
Alopecia	+	+	−	+	−
Rash	−	+	+	+	+
Arthritis	+	+	−	+	+
Myositis	+	+	+	+	−
Antibody					
ANA	+	+	+	+	+
Anti-dsDNA antibody	+	+	+	+	+
Anti-Sm antibody	+	+	−	−	−
Renal pathology	Class IV + V	Class IV + V	Class III + V	Class III + V	Class IV
Concomitant medications before MSCT					
Prednisone dose or equivalent, mg/day	15	5	10	15	15
HCQ, mg/day	300	400	200	400	300

*ANA* anti-nuclear antibody, *anti-dsDNA antibody* anti-double-stranded DNA antibody,

*HCQ* hydroxychloroquine, *MSCT* mesenchymal stem cell transplantation.

### Disruption of CXCL10-CXCR3 axis suppresses hUC-MSCs recruitment to LN kidney

**Fig. 2:**
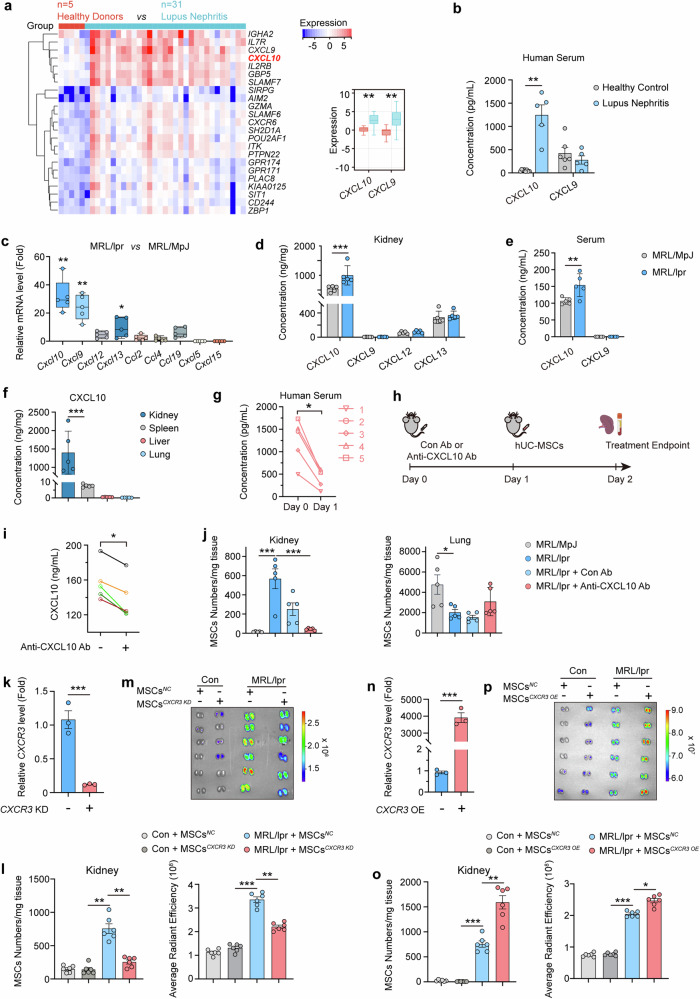
Disruption of CXCL10-CXCR3 axis suppresses hUC-MSCs recruitment to LN kidney. **a** Differential analysis of mRNA expression in the kidneys of LN patients compared to that of healthy donors based on ERCB Lupus TubInt Dataset Summary database. Each square represents one patient. **b** Concentrations of CXCL10 and CXCL9 in serum from LN patients (*n* = 5) and healthy donors (*n* = 6). **c** Relative mRNA expression levels of chemokines are determined by Q-PCR in the kidney of MRL/lpr and MRL/MpJ mice (*n* = 5). **d** The concentrations of chemokines in kidneys from MRL/MpJ and MRL/lpr mice (*n* = 5). **e** The concentrations of CXCL9 and CXCL10 in serum from MRL/MPJ and MRL/lpr mice (*n* = 5). **f** The concentration of CXCL10 in the kidney, spleen, liver, and lung from MRL/lpr mice (*n* = 5). **g** CXCL10 concentrations in the serum of five LN patients in whom treatment was effective before and 1 day after treatment with hUC-MSCs. **h** Scheme illustrating MSC tissue distribution after pretreatment with anti-CXCL10 antibodies in MRL/lpr and MRL/MpJ mice. Mice were pretreated with 20 µg isotype control antibodies or anti-CXCL10 antibodies after 1 day they were injected intravenously with hUC-MSCs. **i** Concentration of CXCL10 in serum from MRL/lpr mice before and after pretreated with anti-CXCL10 antibodies (*n* = 5). **j** Number of hUC-MSCs was determined by Q-PCR in the lungs and kidneys of MRL/lpr and MRL/MpJ mice at 1 day after intravenous injection of hUC-MSCs (*n* = 5). **k** *CXCR3* mRNA expression are measured in hUC-MSCs with or without *CXCR3* knockdown (*n* = 3). hUC-MSCs are transfected with CXCR3 siRNA or negative control (NC) for 24 h, respectively. Each data point represents an independent experiment. **l** Number of hUC-MSCs was determined by Q-PCR in the kidneys of control and MRL/lpr mice at 1 day after intravenous injection of hUC-MSCs^*NC*^ and hUC-MSCs^*CXCR3 KD*^ (*n* = 6). **m** Fluorescence intensity of RFP-hUC-MSCs (the hUC-MSCs expressing RFP fluorescent protein) with or without *CXCR3* knockdown in kidneys is determined by IVIS Imaging System (*n* = 6). **n** *CXCR3* mRNA expression is measured in hUC-MSCs with or without *CXCR3* overexpression (*n* = 3). hUC-MSCs are transfected with *CXCR3* overexpression lentivirus or negative control (NC) for 48 h, respectively. Each data point represents an independent experiment. **o** Number of hUC-MSCs were determined by Q-PCR in the kidneys of control and MRL/lpr mice at 1 day after intravenous injection of hUC-MSCs^*NC*^ and hUC-MSCs^*CXCR3 OE*^ (*n* = 6). **p** Fluorescence intensity of RFP-hUC-MSCs with or without *CXCR3* overexpression in kidneys is determined by IVIS Imaging System (*n* = 6). *P < 0.05, **P < 0.01, ***P < 0.001

Chemokines play a key role in recruiting MSCs to sites of inflammation.^37^ However, it is poorly understood how LN kidneys recruit MSCs. In order to comprehensively assess the variations in chemokine levels within the kidneys affected by LN and determine the specific chemokine signaling pathway responsible for the recruitment of MSCs to diseased kidneys, we conducted a gene analysis of renal tissue obtained from healthy donors and LN patients using the ERCB Lupus Tubert Dataset Summary. The changes of *CXCL10, CXCL9* expression in LN kidneys ranked in the top five of all genes, and both *CXCL10* and *CXCL9* gene levels were significantly higher in kidneys of LN patients than those in healthy controls (Fig. 2a). Meanwhile, in our collected clinical samples, the serum levels of CXCL10 in LN patients were also significantly higher than those in healthy controls while the serum levels of CXCL9 in LN did not differ from those in healthy controls (Fig. 2b). And we also found that the gene levels of *Cxcl10*, *Cxcl9* in the kidneys of MRL/lpr mice were remarkedly increased than MRL/MpJ mice (Fig. 2c). Subsequent protein level detection revealed a significantly elevated concentration of CXCL10 in LN kidneys of MRL/lpr mice compared to the corresponding control group, and CXCL10 was significantly higher than other chemokines such as CXCL9, CXCL12 and CXCL13 (Fig. 2d). In the serum of MRL/lpr mice, CXCL10 levels were significantly higher than that of the control group. However, CXCL9 concentration was much lower than the CXCL10 levels, and there were no obvious changes between the LN and control groups (Fig. 2e). CXCL10 concentrations in different organs were detected, and we found that its levels in kidneys were the highest among all the detected organs (Fig. 2f). Additionally, a significant reduction in serum CXCL10 levels was also observed within 24 h after hUC-MSCs treatment in the five LN patients who responded well to the treatment (Fig. 2g).

To investigate whether CXCL10 can recruit hUC-MSCs to kidneys, we used anti-CXCL10 antibody to block its chemotactic activity in MRL/lpr mice (Fig. 2h). Twenty-four hours after intravenous injection of anti-CXCL10 antibody, the serum levels of CXCL10 in MRL/lpr mice were all decreased (Fig. 2i). So did the CXCL10 levels in the kidneys of MRL/lpr mice (Supplementary Fig. 2a, b). Q-PCR analysis showed that anti-CXCL10 antibody significantly reduced the distribution numbers of MSCs in the kidneys of LN mice. However, anti-CXCL10 antibody did not affect the distribution numbers of MSCs in the lungs (Fig. 2j). Since the corresponding receptor of CXCL10 is CXCR3, we further examined the mRNA and protein levels of CXCR3, CXCR4, and CXCR5 in hUC-MSCs. The expression levels of CXCR3 were much higher than CXCR4 and CXCR5 (Supplementary Fig. 2c). Renal homogenates from MRL/lpr LN mice could stimulate an increase of CXCR3 expression in hUC-MSCs (Supplementary Fig. 2d). To reinforce our conclusions regarding the pivotal role of the CXCL10-CXCR3 axis in directing the renal homing of hUC-MSCs in lupus nephritis, we conducted *CXCR3* knockdown and overexpression manipulations on hUC-MSCs (Fig. 2k, n), followed by a rigorous in vivo analysis of the distribution patterns of these genetically modified MSCs upon intravenous injection. Downregulation of CXCR3 significantly impairs the homing ability of hUC-MSCs to the kidneys of lupus nephritis mice (Fig. 2l, m). Conversely, overexpression of *CXCR3* significantly augments the homing capacity of hUC-MSCs towards the diseased kidneys in lupus nephritis mice (Fig. 2o, p). Furthermore, we examined the effect of CXCL10-CXCR3 axis on the chemotaxis of MSCs by using a transwell system. The addition of either CXCL10 or renal homogenate from LN mice to the lower culture medium significantly increased the numbers of hUC-MSCs migrating from the upper transwell to the lower layer. In contrast, *CXCR3* knockdown MSCs lost the ability to migrate to the lower layer (Supplementary Fig. 2e, f). Similarly, the addition of anti-CXCL10 antibody significantly impaired the effect of MRL/lpr mouse kidney homogenates to recruit hUC-MSCs (Supplementary Fig. 2g). These findings serve to further substantiate the specificity and significance of the CXCL10-CXCR3 axis in facilitating the therapeutic renal homing of hUC-MSCs in lupus nephritis.

### Renal CXCL10 is primarily derived from glomerular vascular endothelial cells via IFN-γ/IFN-γ R pathway in LN

**Fig. 3:**
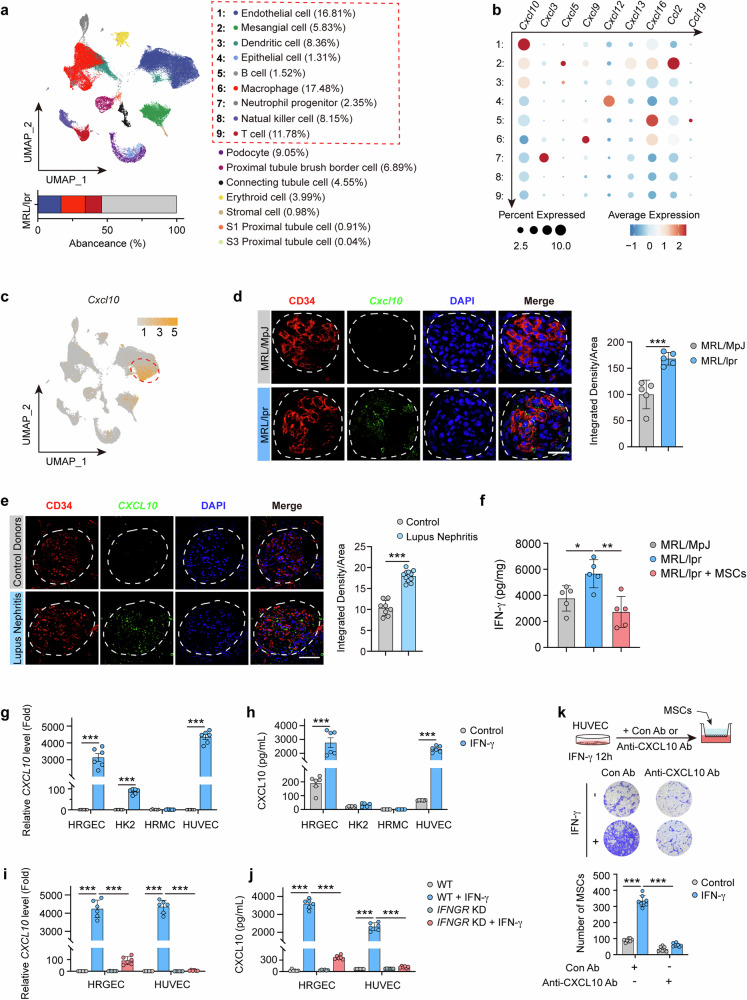
Renal CXCL10 is primarily derived from glomerular vascular endothelial cells via IFN-γ/IFN-γ R pathway in LN. **a** UMAP showing cell clusters from glomerulus of MRL/lpr mice. **b** Air bubble diagram showing mRNA expression of chemokines in cells as indicated. **c** UMAP plot showing expression of *Cxcl10* from glomerulus of MRL/lpr mice. **d** In situ mRNA expression of *Cxcl10* in the glomeruli of MRL/lpr and MRL/MpJ mice. *Cxcl10* in green, CD34 in red (*n* = 5). Scale bar: 20 µm. **e** In situ mRNA expression of *CXCL10* mRNA in the glomeruli of patients with lupus nephritis and controls, *CXCL10* in green, CD34 in red (*n* = 5). Scale bar: 50 µm. **f** Concentration of IFN-γ in kidneys from MRL/MpJ and MRL/lpr mice and MRL/lpr mice at 7 days treated with hUC-MSCs (*n* = 5). **g**, **h** HRGECs, HK-2 cells, HRMCs, and HUVECs were stimulated with or without IFN-γ (50 ng/mL) for 12 h. The mRNA level (**g**) and secretory protein level (**h**) of CXCL10 were detected (*n* = 6). **i**, **j** HRGECs^*NC*^ and HRGECs^*IFNGR KD*^, HUVECs^*NC*,^ and HUVECs^*IFNGR KD*^ were stimulated with or without IFN-γ (50 ng/mL) for 12 h. The mRNA level (**i**) and secretory protein level (**j**) of CXCL10 were detected (*n* = 6). **k** Representative images and numbers of hUC-MSCs recruited by culture medium supernatants which were collected after 12 h of IFN-γ (50 ng/mL) stimulation of HUVECs and with or without anti-CXCL10 antibody (5 µg/mL) (*n* = 3). Cell migration was assessed by transwell assays. Data represent mean ± SD. *P < 0.05, **P < 0.01, ***P < 0.001

Our above data showed hUC-MSCs recruitment to LN kidneys primarily distributed in glomeruli, and the pathological changes were mainly reflected by the improvement of glomerulosclerosis. Therefore, we performed single-cell RNA sequencing after separating the glomeruli of MRL/lpr LN mice with or without MSC treatment (Supplementary Fig. 3a, b). Based on the UMAP plots of glomerular sequencing results and the percentage of individual cell lineages to the total number of cells, we found that endothelial cells, macrophages, and T cells were the three most abundant cell types in the glomeruli of MRL/lpr mice (Fig. 3a). Marker genes for each cell cluster are shown in Supplementary Fig. S3b. We further conducted a comprehensive analysis of the gene levels of chemokines known to recruit MSCs in glomerular intrinsic cells. Our findings indicated that endothelial cells exhibited significantly higher levels of *Cxcl10* compared to other chemokines. *Cxcl10* expression in endothelial cells was also significantly higher than that in other cell lines (Fig. 3b). UMAP plots depicting the expression distribution of *Cxcl10* in the glomeruli of MRL/lpr mice further support the predominant expression of *Cxcl10* in endothelial cells (Fig. 3c). Additionally, RNAscope revealed the presence of fluorescent-labeled *Cxcl10* mRNA primarily in the glomeruli and colocalization with vascular endothelial cells in the kidneys of MRL/lpr mice (Fig. 3d). Renal puncture samples from LN patients and controls also revealed that the fluorescent signals of *CXCL10* mRNA were predominantly localized in the glomeruli of LN patients, with a dominant colocalization of *CXCL10* signals with vascular endothelial cells in the glomeruli (Fig. 3e).

IFN-γ plays a key role in inducing CXCL10 production and promoting SLE disease progression.^38^ We used RNA sequencing (RNA-seq) analysis to compare the transcriptomes of interferon-responsive (IFN-γ high) SLE, interferon-non-responsive (IFN-γ low), and healthy control (HC) human PBMC samples from the GEO database. RNA-seq results of PBMC showed higher *CXCL10* expression in IFN-γ high SLE compared to IFN-γ low group or HC (Supplementary Fig. 3c). Consistently, IFN-γ levels in serum (Supplementary Fig. 3d) and kidney (Fig. 3f) of MRL/lpr mice were significantly higher than that of MRL/MpJ mice. To investigate whether CXCL10 is mainly derived from glomerular endothelial cells when the circumstance contains a high level of IFN-γ, human renal glomerular endothelial cells (HRGECs), epithelial cells (HK2 cells), human renal thylakoid cells (HRMCs) and human umbilical vein endothelial cells (HUVECs) were used. Both *CXCL10* mRNA levels in the cells and CXCL10 concentration in the supernatant of cell culture showed that IFN-γ induced a significant increase of CXCL10 production in HRGECs and HUVECs, which was much higher than that in HK2 and HRMCs (Fig. 3g, h). The production of CXCL10 remained at a very low level in both epithelial and thylakoid cells. *IFNGR* knockdown significantly inhibited the production of CXCL10 in HRGECs and HUVECs (Fig. 3i, j and Supplementary Fig. 3e). In addition, the recruitment of hUC-MSCs from the upper transwell to the lower layer that incubated with the supernatant of HUVEC after IFN-γ stimulation was also blocked by anti-CXCL10 antibody (Fig. 3k). Addition of IFN-γ and anti-CXCL10 antibody to blank HUVEC medium did not affect its recruiting ability towards hUC-MSCs (Supplementary Fig. 3f). Furthermore, IFN-γ induced a significant increase of CXCL10 production in HRGECs than that in M0, M1 and M2 macrophages (Supplementary Fig. 3g–j), and IFN-γ induces CXCL10 production in endothelial cells more potently than did the anti-dsDNA antibody (Supplementary Fig. 3k, l). Collectively, CXCL10 in LN kidneys is mainly produced by glomerular vascular endothelial cells, and IFN-γ/IFN-γ R pathway mainly contributed to the production of CXCL10 in glomerular vascular endothelial cells.

### Nuclear transport of IRF1-KPNA4 mediates IFN-γ-induced secretion of CXCL10 from endothelial cells

**Fig. 4:**
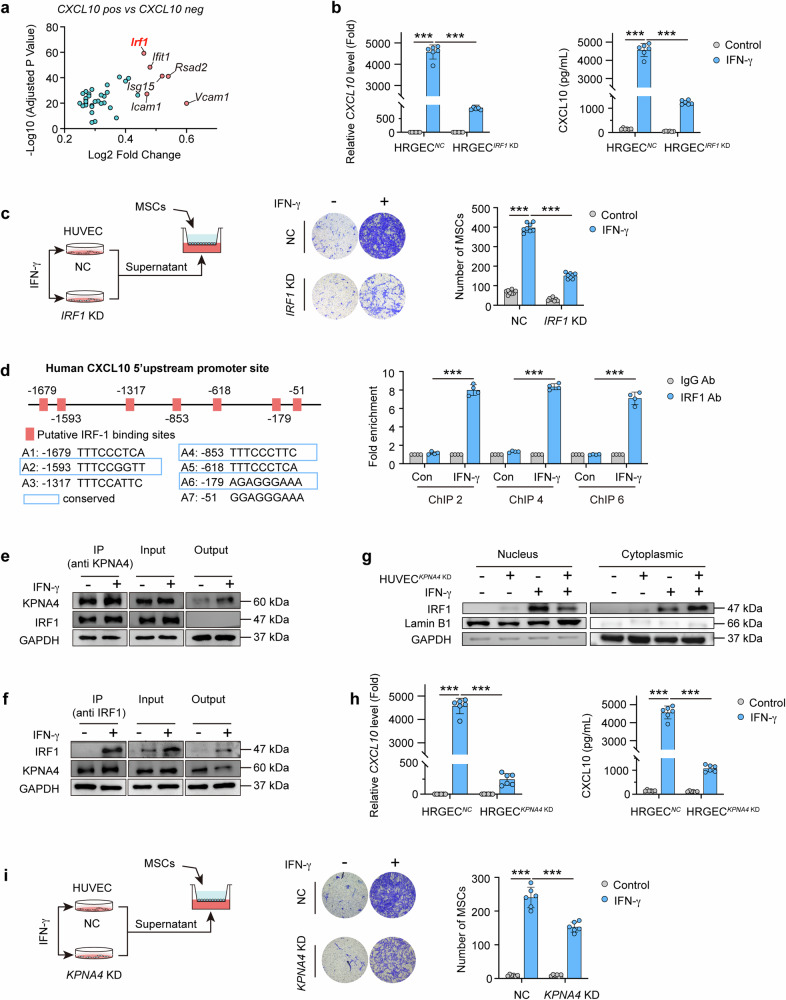
Nuclear transport of IRF1-KPNA4 mediates IFN-γ-induced secretion of CXCL10 from endothelial cells. **a** Fold change in gene expression levels of *Cxcl10* positive expressing endothelial cells compared to *Cxcl10* negative expressing endothelial cells from single-cell sequencing of MRL/lpr mouse glomeruli. **b** HRGECs^*NC*^ and HRGECs^*IRF1 KD*^ were stimulated with or without IFN-γ (50 ng/mL) for 12 h. The mRNA level and secretory protein level of CXCL10 were detected (*n* = 6). **c** Representative images and numbers of hUC-MSCs recruited by culture medium supernatants collected after 12 h of IFN-γ (50 ng/mL) stimulation in HUVECs^*NC*^ or HUVECs^*IRF1 KD*^ (*n* = 3), cell migration was assessed by transwell assays. **d** Schematic representation of IRF1 binding sites in the human CXCL10 promoter region as predicted by PROMO bioinformatics software. ChIP assay is performed with IgG or anti-IRF1 antibody in cell lysis from HUVEC cells which are induced by human IFN-γ (50 ng/mL) for 8 h. The Q-PCR analysis of immunoprecipitated DNA is conducted using the primers which are designed to amplify the indicated region of the CXCL10 promoter. Each data point represents an independent experiment (*n* = 3). **e**, **f** The lysates of HUVEC cells with or without human IFN-γ (50 ng/mL) for 8 h were subjected to immunoprecipitation (IP) using anti-KPNA4 (**e**) antibody or anti-IRF1 antibody (**f**) and the enriched proteins were identified by western blotting to determine the protein level of IRF1 (**e**) or KPNA4 (**f**). **g** Total protein, cytoplasmic protein, and nucleoprotein expression of IRF1 are respectively analyzed by western blotting in HUVECs^*NC*^ and HUVECs^*KPNA4 KD*^ cells with or without human IFN-γ (50 ng/mL) for 8 h. **h** HRGECs^*NC*^ and HRGECs^*KPNA4 KD*^ were stimulated with or without IFN-γ (50 ng/mL) for 12 h. The mRNA level and secretory protein level of CXCL10 were detected (*n* = 6). **i** Representative images and number of hUC-MSCs recruited by culture medium supernatants which were collected after 12 h of IFN-γ (50 ng/mL) stimulation of HUVECs^*NC*^ or HUVECs^*KPNA4 KD*^ (*n* = 3). Cell migration was assessed by transwell assays. HRGECs or HUVECs are transfected with *IRF1* or *KPNA4* siRNA or negative control (NC) for 24 h, respectively. Data represent mean ± SD. ***P < 0.001

To further investigate the mechanism by which IFN-γ induces endothelial cells to secrete CXCL10. UMAP plots of *Cxcl10* in the glomerular single-cell sequencing results of MRL/lpr mice showed that *Cxcl10* expression was not equally distributed in vascular endothelial cells. So, the glomerular vascular endothelial cells of MRL/lpr mice were divided into two group: *Cxcl10*-positive and *Cxcl10*-negative groups. Among the significantly upregulated genes between the two groups, *Irf1, Rsad2*, *Ifit1*, and *Isg15*, which were the downstream genes of interferon, ranked in the top ten (Fig. 4a). IRF1, a transcription factor that has been reported to promote the expression of CXCL10 in HCC (Hepatoma carcinoma cell) cells,^39^ was found a high expression level in glomerular vascular endothelial cells (Supplementary Fig. 4a). In contrast, the other two transcription factors *Stat1* and *Foxp1* related to the regulation of CXCL10 were barely expressed in endothelial cells (Supplementary Fig. 4a). Meanwhile, there was almost no change in *Stat1* and *Foxp1* levels in endothelial cells after treatment with hUC-MSCs in MRL/lpr mice (Supplementary Fig. 4b). We then examined the effect of *IRF1* knockdown on CXCL10 production in HRGECs and HUVECs after stimulation with IFN-γ. *IRF1* knockdown significantly impaired the ability of endothelial cells to produce and secret CXCL10 after stimulation with IFN-γ (Fig. 4b and Supplementary Fig. 4c–e). In addition, *IRF1* knockdown in HUVECs significantly impaired the recruitment to hUC-MSCs co-cultured with their medium supernatants 12 h after IFN-γ stimulation (Fig. 4c). The above results suggested that IRF1 might play an important role in IFN-γ induced CXCL10 production in glomerular endothelial cells. The fact that IRF1 is a transcription factor and that *CXCL10* mRNA levels were modulated led us to examine whether IRF1 could act as a direct activator of *CXCL10* transcription. In silico analysis of the CXCL10 promoter region identified seven putative IRF1 responsive elements (A1-A7). Subsequently, we performed chromatin immunoprecipitation (ChIP) in cell lysates of HUVEC cells with anti-IRF1 antibody and PCR primers spanning each responsive element. IRF1 binding to the *CXCL1*0 promoter construct (A2: −1593 region, A4: −853 region, and A6: −179 region) induced by IFN-γ was observed, whereas transactivation was not detected on the other putative ISRE elements (Fig. 4d).

In endothelial cells, IRF1 proteins are mainly found in the nucleus (Supplementary Fig. 4f). To explore which protein that maintains the nuclear localization of IRF1. LC-MS analysis with Co-IP samples using anti-IRF1 antibody found that the nuclear transporter protein, KPNA4, showed a high degree of binding to IRF1 protein (Supplementary Fig. 4g). Then, Co-IP with anti-IRF1 antibody and anti-KPNA4 antibody respectively in HUVECs cell lyses showed substantial binding of IRF1 protein and KPNA4 protein in endothelial cells after IFN-γ stimulation (Fig. 4e, f). The nuclear localization of both intracellular KPNA4 and IRF1 was increased in HUVECs after IFN-γ stimulation (Supplementary Fig. 4h). *KPNA4* knockdown decreased nuclear localization of IRF1 in IFN-γ-induced HUVECs (Fig. 4g and Supplementary Fig. 4i). Furthermore, *KPNA4* knockdown remarkably reduced the production and secretion of CXCL10 in HRGECs and HUVECs stimulated with IFN-γ (Fig. 4h and Supplementary Fig. 4j-l). Similarly, medium supernatants of *KPNA4* knockdown HUVECs exhibited an impaired ability to drive the recruitment of hUC-MSCs after IFN-γ stimulation (Fig. 4i).

### Amelioration of endothelial inflammation in glomeruli of lupus nephritis by hUC-MSCs

**Fig. 5:**
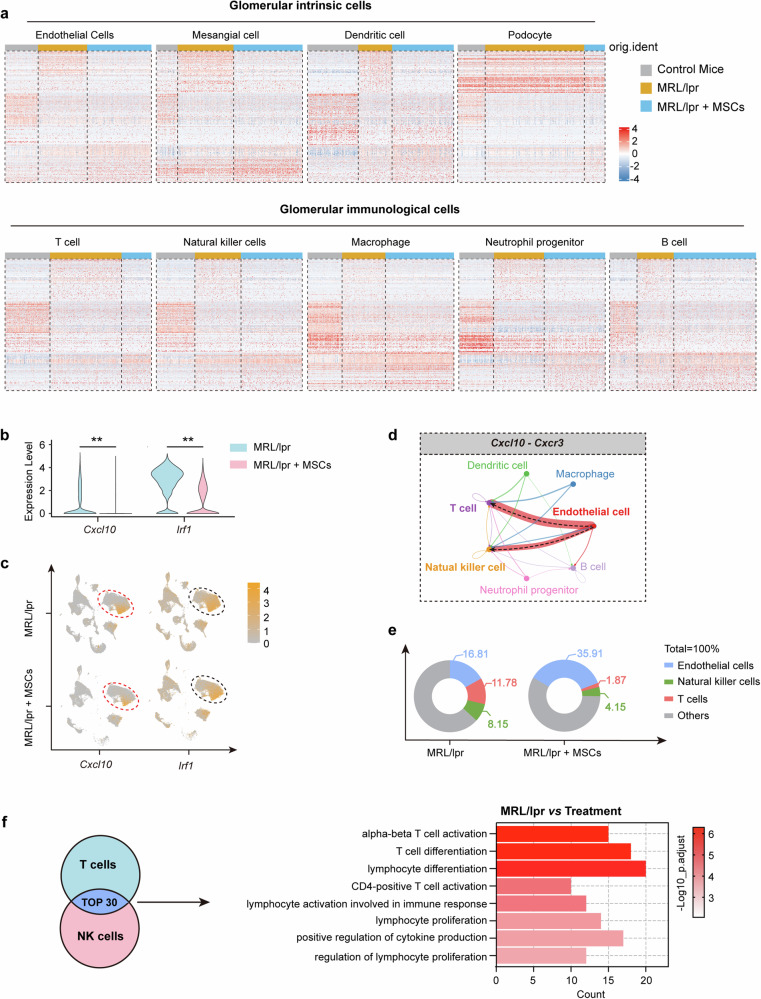
Amelioration of endothelial inflammation in glomeruli of lupus nephritis by hUC-MSCs. **a** Heatmaps showing the top variably expressed genes in glomerular intrinsic cells and glomerular immunological cells of control mice, MRL/lpr mice and MRL/lpr mice treated with hUC-MSCs. Each column represents a cell, each row represents a gene. **b**, **c** Violin diagram (**b**) and UMAP plot (**c**) of *Cxcl10* and *Irf1* gene levels in glomerular endothelial cells of MRL/lpr mice at 21 days treated with or without hUC-MSCs. **d** Cell chat analysis between endothelial cells and immune cells of MRL/lpr mouse glomeruli through the Cxcl10-Cxcr3 axis. **e** Distribution of the percentage of cells, as indicated in glomeruli of MRL/lpr mice at 21 days, treated with or without hUC-MSCs. **f** Inflammatory pathway activation levels of T cells and NK cells in glomeruli of MRL/lpr mice at 21 days treated with or without hUC-MSCs. Data represent mean ± SD. **P < 0.01, ***P < 0.001

The above data showed the inhibitory effect on IFN-γ-induced endothelial CXCL10 secretion by MSCs. So, we performed an analysis on the top variably expressed genes in both intrinsic glomerular cells and glomerular immune cells of control mice, MRL/lpr mice and MRL/lpr mice treated with hUC-MSCs by sc-RNAseq. Heatmap showed that genes were significantly changed in intrinsic and immune cells in glomeruli of MRL/lpr mice, especially the inflammatory pathways in endothelial cells, T cells, and NK cells (Fig. 5a). Furthermore, the levels of interferon-related genes (*Cxcl10*, *Irf1*) in glomerular endothelial cells were significantly lower in MRL/lpr treated with hUC-MSCs than that in untreated MRL/lpr mice (Fig. 5b, c). In addition, the changing trend of other interferon-related genes (*Ifit1*, *Rsad2*, *Isg15*) and lymphatic cell infiltration-associated endothelial adhesion molecule (*Icam1*) in glomerular endothelial cells is consistent with *Irf1* (Supplementary Fig. 5). Cell chat analysis revealed that *Cxcl10-Cxcr3* axis primarily governed the communication between endothelial cells and T cells/ NK cells. And the cell chat between endothelial cells and T cells displayed the highest communication degree (Fig. 5d). To further investigate the effect of hUC-MSCs treatment on the immune microenvironment, we analyzed the percentage of T cells, NK cells, and endothelial cells to the total cell count in the glomeruli of MRL/lpr mice. Compared with untreated MRL/lpr mice, the percentage of T cells and NK cells to total cells was significantly reduced (T cells: from 11.78 to 1.87%, NK cells: from 8.15 to 4.15%), and the percentage of endothelial cells to total cells was increased (from 16.81 to 35.91%) in glomeruli of MRL/lpr mice treated with hUC-MSCs (Fig. 5e). Then, the changes in sc-RNAseq gene pathways of T cell and NK cell showed that T cell and NK cell inflammatory pathway (lymphocyte proliferation, differentiation, and activation-related pathways) activation levels were reduced in glomeruli of MRL/lpr mice treated with hUC-MSCs compared with the untreated MRL/lpr mice (Fig. 5f).

### hUC-MSCs reduce the infiltration of CXCR3^+^ Th1 cells into lupus nephritis kidneys

**Fig. 6:**
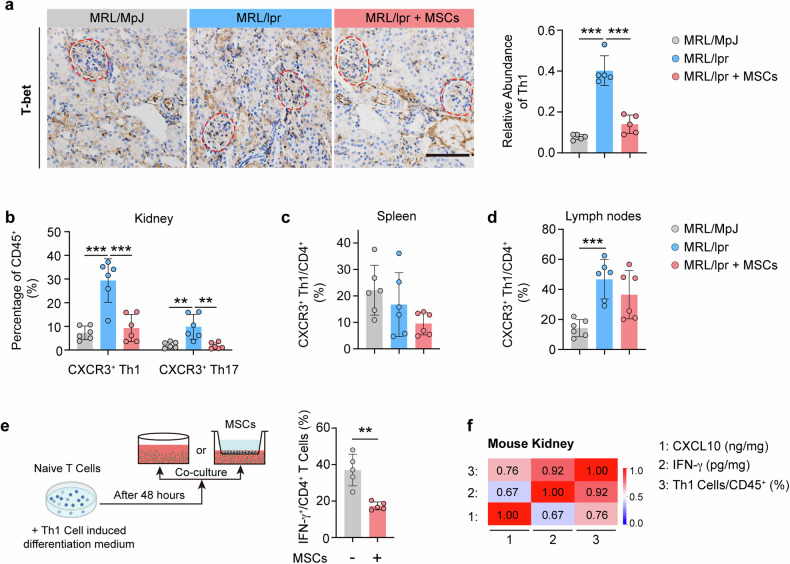
hUC-MSCs reduce the infiltration of CXCR3^+^ Th1 cells into lupus nephritis kidney. **a** Number of Th1 cells in glomeruli of MRL/MpJ and MRL/lpr mice at 21 days treated with or without hUC-MSCs (*n* = 6). Th1 cells were detected using T-bet immunohistochemical labeling. Scale bar: 50 µm. **b** Frequencies of CXCR3^+^ Th1/CD45^+^ and CXCR3^+^ Th17/CD45^+^ cells in kidneys from MRL/MpJ mice and MRL/lpr mice at 7 days treated with or without hUC-MSCs were detected by flow cytometry (*n* = 6). **c**, **d** Frequencies of CXCR3^+^ Th1/CD4^+^ cells in the spleen (**c**), lymph nodes (**d**) from MRL/MpJ mice and MRL/lpr mice at 7 days treated with or without hUC-MSCs were detected by flow cytometry (*n* = 6). **e** Representative flow cytometry plots and frequencies of CD4^+^ IFN-γ^+^/CD4^+^ cells in Th1 cells with or without Co-culture with hUC-MSCs by transwell (*n* = 5). **f** Heatmap of the correlation between protein levels of CXCL10, IFN-γ and the frequencies of Th1 cells in the kidney of MRL/lpr mice, respectively. Data represent mean ± SD. **P < 0.01, ***P < 0.001

The increased infiltration of renal T cells, particularly Th1 cells, emerged as a significant pathological alteration in LN. Subsequently, we focused on studying Th1 cells in LN kidneys. T-bet immunohistochemical staining showed that there was a significant increase in the numbers of Th1 cells infiltrating (Brown, rounded, smaller cells) into the glomeruli of MRL/lpr mice than that in MRL/MpJ mice, and this was reversed by the treatment of hUC-MSCs (Fig. 6a). Then, flow cytometry was used to detect the presence of CXCR3^+^ Th1 and CXCR3^+^ Th17 cell subpopulations in the kidneys, spleens, and lymph nodes of MRL/lpr mice (Supplementary Fig. 6a). The ratios of CXCR3^+^ Th1 cells/CD45^+^ cells and CXCR3^+^ T17 cells/CD45^+^ cells in the kidneys of MRL/lpr mice were all found to be significantly higher than those in MRL/MpJ mice. However, treatment with hUC-MSCs inhibited these ratios (Fig. 6b). Furthermore, within the CXCR3^+^ T cell population, the majority consisted of CXCR3^+^ Th1 cells, while CXCR3^+^ Th17 cells represented a smaller proportion (Fig. 6b). However, there was no statistically significant disparity in the ratios of CXCR3^+^ Th1 cells/CD4^+^ cells in the lymph nodes and spleens between MRL/lpr mice treated with hUC-MSCs and those untreated (Fig. 6c, d). The elevated levels of IFN-γ and CXCL10 in MRL/lpr kidneys were also alleviated after administration of hUC-MSCs (Supplementary Fig. 6b, c).

To investigate whether MSCs inhibit the activation of Th1 cells through the secretion pathway, we employed a transwell system to co-culture the two cell types with physical separation. The results showed that hUC-MSCs significantly reduced the ability of Th1 cells to produce IFN-γ (Fig. 6e). Finally, we did a correlation analysis between CXCL10, IFN-γ levels, and the number of Th1 cells infiltrated in the kidneys of MRL/lpr mice, respectively. CXCL10, IFN-γ levels, and the number of Th1 cells in the kidneys of mice were positively correlated, respectively (Fig. 6f). The above results suggest that hUC-MSCs treatment reduces the number of CXCR3^+^ Th1 cell infiltration in LN kidneys by attenuating interferon production.

### IL4I1 derived from hUC-MSCs inhibits renal CXCL10 level and the infiltration of CXCR3^+^ Th1 cell in LN

**Fig. 7:**
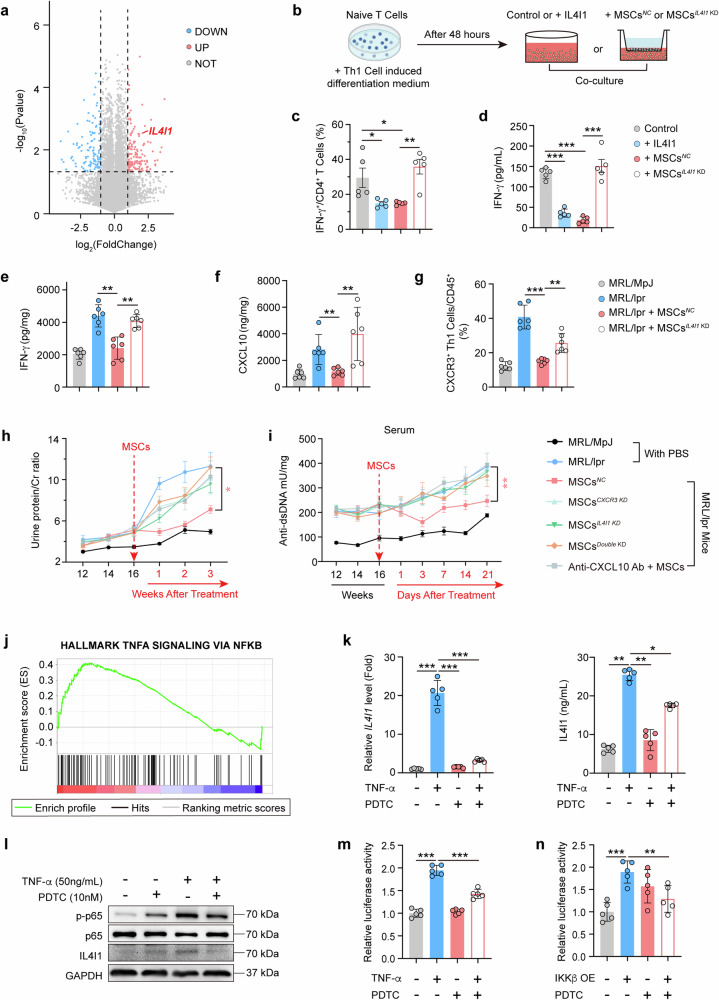
IL4I1 derived from hUC-MSCs inhibits renal CXCL10 level and the infiltration of CXCR3^+^ Th1 cell in LN. **a** Genome sequencing of hUC-MSCs with renal homogenate stimulation for 8 h in MRL/lpr and MRL/MpJ mice and analysis of differential genes in the MRL/lpr group relative to the MRL/MpJ group. **b** Schematic diagram of the method for co-culturing mouse Th1 cells with hUC-MSCs, and flow assay strategy for IFN-γ^+^ immune cells. **c** IL4I1 functional protein was added to the culture medium or co-culturing hUC-MSCs^*NC*^, and hUC-MSCs^*IL4I1 KD*^ with Th1 cells, respectively. And frequencies of IFN-γ^+^/CD4^+^ cells were detected by flow cytometry after 72 h of culture (*n* = 5). **d** Concentration of IFN-γ in Th1 cell culture medium supernatant in (**c**) (*n* = 5). **e**, **f** IFN-γ (**e**) and CXCL10 (**f**) concentrations in the kidneys of MRL/MpJ or MRL/lpr mice at 7 days treated with or without hUC-MSCs^*NC*^ (Negative Control) or hUC-MSCs^*IL4I1 KD*^ (*IL4I1* knockdown). **g** The frequencies of CXCR3^+^ Th1/CD45^+^ cells in kidneys from mice of (**e**) were detected by flow cytometry (*n* = 6). **h** Dynamic curves of urine protein/creatinine levels in MRL/MpJ and MRL/lpr mice before and after treatment of hUC-MSCs with different gene-knockdown phenotypes (*n* = 5). **i** Dynamic curves of serum anti-dsDNA antibody levels in MRL/MpJ and MRL/lpr mice before and after treatment of hUC-MSCs with different gene-knockdown phenotypes (*n* = 5). **j** Enrichment analysis of IL4I1-related pathways in (**a**) genome sequencing result. **k** hUC-MSCs were pretreated with DMSO or PDTC (10 µM) for 2 h and then stimulated with or without TNF-α (50 ng/mL) for a further 4 h. The mRNA level and secretory protein level of IL4I1 were detected (*n* = 5). **l** hUC-MSCs were treated as in (**k**), cell lysates were collected and protein expression levels of p-p65, p65, and IL4I1 were respectively measured by western blotting. p65 and GAPDH as internal reference for p-p65 and IL4I1, respectively. **m** HEK293T cells were pretreated with DMSO or PDTC (10 µM) for 2 h and then treated with or without TNF-α (50 ng/mL) for 4 h, followed by a luciferase reporter gene assay to examine the luciferase activity of the transcription factor RelA and IL4I1 promoter binding in HEK293T cells (*n* = 5). **n** Luciferase reporter gene assay to examine the luciferase activity of transcription factor RelA and IL4I1 promoter binding in HEK293T cells after overexpression of *IκKβ* and pretreatment with DMSO or PDTC (10 µM) for 2 h (*n* = 5). Data represent mean ± SD. *P < 0.05, **P < 0.01, ***P < 0.001

To investigate which key immunoregulatory factor of hUC-MSCs may respond to the renal environment of LN, RNAseq analysis of hUC-MSCs after co-cultured with renal homogenates from MRL/lpr or MRL/MpJ mice was conducted. The mRNA levels of *IL4I1*, considered as a key immunoregulator at a crossroads of divergent T-cell functions, were found significantly elevated in hUC-MSCs after stimulation with renal homogenate from MRL/lpr LN mice compared to those of MRL/MpJ group (Fig. 7a). Single-cell sequencing analysis of glomerular in MRL/lpr mice reveals minimal expression of *IL4I1* within macrophages and other renal cells (Supplementary Fig. 7a). Both IL4I1 and co-culture with hUC-MSCs markedly reduced the ratios of IFN-γ producing T cells and IFN-γ secretion in the culture medium, and the effects of hUC-MSCs was reversed by *IL4I1* knockdown (Fig. 7b–d). Then, the effects of IL4I1 derived from hUC-MSCs were validated in vivo. MRL/lpr mice treated with hUC-MSCs^*IL4I1 KD*^ showed weaker effects than hUC-MSCs^*NC*^ on IFN-γ concentration, CXCL10 level, and CXCR3^+^ Th1 cell number in the kidneys (Fig. 7e–g and Supplementary Fig. 7b), suggesting a critical role of IL4I1 from hUC-MSCs in regulating the infiltration of CXCR3^+^ Th1 cells into the kidneys of LN mice. However, the numbers of CXCR3^+^ Th1 cells in the spleen and lymph nodes of MRL/lpr mice treated with hUC-MSCs^*NC*^ or hUC-MSCs^*IL4I1 KD*^ did not differ significantly compared with MRL/lpr mice (Supplementary Fig. 7c). To further comprehensively examine the effects of the CXCL10-CXCR3 axis and the production of IL4I1 on the treatment of lupus nephritis with hUC-MSCs, we used individual knockdown and double knockdown strategies for CXCR3 and IL4I1 (Supplementary Fig. 7d, e), and a strategy of in vivo neutralization with Anti-CXCL10 Ab for CXCL10 (Supplementary Fig. 7f). The findings revealed a continuous escalation in the levels of urine protein/creatinine and serum anti-dsDNA antibody in the MRL/lpr mice. However, this escalation was effectively attenuated in the group treated with hUC-MSCs^*NC*^. Furthermore, the capacity of hUC-MSCs with *CXCR3* knockdown (KD), *IL4I1* KD, and double KD to mitigate the elevated levels of urine protein/creatinine, and serum anti-dsDNA antibody levels in the MRL/lpr mice was significantly impaired (Fig. 7h, i). In comparison to MRL/lpr model mice, the injection of hUC-MSCs^*NC*^ significantly attenuated pathological injury, immune complex IgG deposition, spleen weight, and skin injury after 21 days of treatment. Conversely, the mitigating effects of hUC-MSCs^*CXCR3 KD*^, hUC-MSCs^*IL4I1 KD*^ and hUC-MSCs^*Double KD*^ treatment on the aforementioned indicators were significantly less potent than those of hUC-MSCs^*NC*^ (Supplementary Fig. 7g–j). The above results elucidate the pivotal roles of CXCL10-CXCR3 axis and IL4I1 in MSC-mediated therapy for LN.

To explore the underlying mechanisms of IL4I1 production in hUC-MSCs, pathway enrichment analysis of the genome sequencing was conducted. TNFA-NFΚB pathway was found extensively activated in hUC-MSCs after stimulation with renal homogenates from MRL/Lpr LN mice (Fig. 7j). Meanwhile, TNF-α levels in the serum of MRL/lpr mice were considerably higher than that of MRL/MpJ mice (Supplementary Fig. 7k). And following treatment with hUC-MSCs, a reduction in serum TNF-α concentrations was also observed in the five lupus nephritis patients who responded well to the treatment (patients 1 to 5) (Supplementary Fig. 7l). The IL4I1 mRNA and protein significantly upregulated by TNF-α (Fig. 7k). To assess whether TNF-α promotes IL4I1 expression through NF-κB, we conducted western blotting experiments and observed an increase in p-p65 expression induced by TNF-α (Fig. 7l). Next, we constructed a luciferase reporter plasmid containing the IL4I1 promoter region, found that TNF-α stimulation significantly enhanced IL4I1 promoter transactivation (Fig. 7m). PDTC (Pyrrolidinedithiocarbamate), a small-molecule inhibitor of NF-κB, can significantly reverse the TNF-α-induced expression of IL4I1 at the gene level, protein level, and promoter transactivation (Fig. 7l–n). Similarly, IL4I1 promoter transactivation was significantly upregulated by overexpression of *IKKβ*, but not PDTC treatment (Fig. 7n and Supplementary Fig. 7m). Our results suggest that IL4I1 is an important factor induced by TNF-α in MSCs, which can effectively inhibit the activation of Th1 cells. To further clarify the key role of hUC-MSCs in the production of IL4I1, we examined the production of IL4I1 in macrophage cells (Supplementary Fig. 8a). It was also found that the tissue spaces in the lungs of MRL/lpr mice were larger than those in control mice, which partly explains why the number of hUC-MSCs distributed in the lungs of MRL/lpr mice was lower than that in control mice (Supplementary Fig. 8b).

## Discussion

**Fig. 8:**
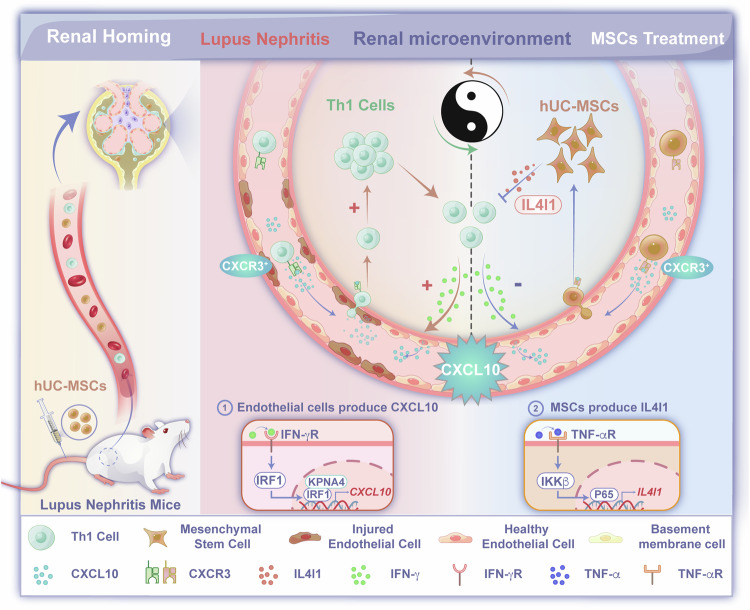
hUC-MSCs are recruited to LN kidneys via the CXCL10-CXCR3 axis and subsequently the production of IL4I1 to inhibit the infiltration of CXCR3^+^ Th1 cells. The significantly increased levels of IFN-γ stimulate glomerular vascular endothelial cells to produce CXCL10, which facilitates the infiltration of CXCR3^+^ Th1 cells into kidneys, thereby aggravating the progression of disease. Interestingly, when hUC-MSCs are administered intravenously, they can be activated through the CXCL10-CXCR3 axis, leading to their migration to LN kidneys. Subsequently, hUC-MSCs exert an inhibitory effect on the infiltration of CXCR3^+^ Th1 cells by TNF-α-induced secretion of IL4I1 and contribute to the restructure of the renal immune microenvironment

To clarify the relationship between the distribution of MSCs in vivo and the improvement of renal function, the present study started with the spatiotemporal characteristics of kidney distribution after MSCs homing and conducted a thorough analysis of the interaction between MSCs and kidney host cells. We demonstrated that the CXCL10-CXCR3 axis played a crucial role in mediating the homing of MSCs to the LN kidney, with CXCL10 primarily originating from glomerular vascular endothelial cells through the INF-γ-IRF1/KPNA4 pathway. Notably, the homing of MSCs to the nephritic kidney resulted in a reduction in the infiltration of CXCR3^+^ Th1 cells through the secretion of IL4I1 and subsequently improved the immune microenvironment in LN. This study revealed the mechanisms of MSC homing and their roles in restoring glomerular homeostasis of the immune microenvironment (Fig. 8).

To date, the variability of MSCs efficacy has been observed in many clinical trials among individuals, so their successful translation to clinical use as advanced medicinal products requires a detailed understanding of MSCs biodistribution, their crosstalk with the host cells and the ultimate fate. The homing capacity of MSCs is often limited when administered systemically, resulting in only a small fraction of cells reaching the target tissue.^17,40^ Currently, the CXCL12-CXCR4 axis is extensively investigated in various medical conditions such as myocardial infarction, colitis, acute respiratory distress syndrome, kidney ischemia/reperfusion injury, and liver injury.^19,21,24,41,42^ However, overexpression of CXCR4 and CXCR7 did not result in improved MSC homing and therapeutic potentials in experimental acute kidney injury, which has sparked controversy.^26^ Several reports have indicated the absence of CXCR4 expression in MSCs,^27^ and so did our results by western blotting. A published study discovered that *CXCR3* knockdown decreased the infiltration of mice-derived BM-MSCs into the nephritic kidneys.^43^ Our data strongly demonstrated for the first time that the diseased status of LN kidney could dramatically increase the numbers of recruited human-derived MSCs to kidneys (4.7-folds of control group) by CXCL10-CXCR3 axis. Consequently, the mechanisms underlying MSC homing may differ depending on the cell source or specific disease microenvironment. Elucidating the distinct regulatory mechanisms of homing in specific diseases holds significance for the clinical utilization of MSCs.

CXCL10, also known as interferon-γ-inducible protein 10 (IP-10), has emerged as a noteworthy and promising biomarker for assessing the severity of renal diseases, including renal allograft dysfunction and LN.^29,44,45^ Previous studies have reported that CXCL10 expression can be induced in various cell types, such as mesangial cells, tubular epithelial cells, podocytes, and endothelial cells in a stimulus-specific manner.^29^ In our present study, the cellular origins of intrarenal CXCL10 and the regulatory mechanisms in LN were clarified thoroughly. By employing single-cell sequencing techniques on isolated glomerular cells from MRL/lpr LN mice, it was discovered that *Cxcl10* primarily originates from glomerular endothelial cells rather than mesangial cells, tubular epithelial cells, or other cell types. A recent report has indicated that the transcription factor Fli-1 (a member of the ETS family of transcription factors) affects CXCL10 production in LN kidneys through indirect regulation.^33^ It has also been documented that Fli-1 mediates the expression of CXCL13 in the kidneys of MRL/lpr mice.^46^ However, our study provide evidence for the pivotal role of IFN-γ−IRF1 pathway in the production of CXCL10 in glomerular endothelial cells of LN. Among the top genes associated with complete clinical response clustering in kidney biopsies from LN patients are IRF1, STAT1, IRF7, MX1, STAT2, and JAK2.^47^ Many studies have demonstrated the necessity of nuclear translocation of mammalian IRF1 for its regulatory effects on downstream genes.^48^ However, the mechanism by which IRF1 translocates to the nucleus in glomerular endothelial cells and maintains its nuclear distribution remains unclear.

IRF1, an interferon regulatory factor, plays a crucial role in immune responses.^48–50^ Many studies have demonstrated the necessity of nuclear translocation of mammalian IRF1 for its regulatory effects on downstream genes.^48^ However, the mechanisms by which IRF1 translocates to the nucleus in glomerular endothelial cells and maintains its nuclear distribution remain unclear. Stimulation with IFN-γ for 24 h has been found to enhance the physical binding of KPNA2 to IRF1 in epidermal keratinocytes (NHEKs) and induce nuclear translocation of IRF1 in NHEKs.^51^ Our study found that in IFN-γ-stimulated endothelial cells, binding of KPNA4 (not KPNA2) to IRF1 was increased and mediated nuclear translocation of IRF1. Our study reported for the first time that KPNA4-IRF1 binding and subsequent translocation to nuclei mediated the production of CXCL10 induced by IFN-γ in LN.

Our findings from single-cell sequencing have revealed that the CXCL10-CXCR3 axis governs the communication between endothelial cells and T cells/NK cells. And MSCs treatment alleviated the injury of glomerular endothelial cells and reduced the infiltration of T cells/NK cells. This prompts the question of how the recruited MSCs impact the immune microenvironment of LN. Previous studies have reported that the infiltration of T cells into the kidneys is a characteristic feature of LN in both human and experimental mice, and this infiltration contributes to the development of renal damage.^52–54^ The expression of CXCR3 was found to be significantly elevated on Th1 cells in some immune diseases, playing a crucial role in their migration into inflamed tissues.^55^ In renal biopsies of LN patients, an average of 63% of infiltrating immune cells expressed CXCR3, with approximately 60% being T cells.^38^ Our study provides solid evidence that hUC-MSCs treatment significantly reduced the infiltration of CXCR3^+^ T cells into LN kidneys as well as the levels of IFN-γ and CXCL10 within the renal tissues. Here, we proposed that CXCR3^+^ T cells can be recognized as a crucial subset of T cells that potentially contribute to the pathogenesis of LN and serve as an indicator of therapeutic efficacy.

The pathway responsible for mediating the remodeling of the immune microenvironment in the kidneys of LN after MSC homing remains unknown. It has been extensively documented that MSCs can exert immunosuppressive effects by secreting soluble proteins (such as PGE2, IDO, TGF-β, NO, etc.) in a paracrine manner, thereby reducing the excessive activation of the immune system.^56–58^ Interestingly, our research revealed a significant upregulation of IL4I1 in hUC-MSCs and the crucial roles in the immune regulation in Th1 cells. IL4I1, also known as interleukin 4 inducible gene 1, is an enzyme that converts phenylalanine to phenylpyruvate, hydrogen peroxide, and ammonia.^59^ Increasing studies have shown that IL4I1 can be considered as a metabolic immune checkpoint.^60^ And IL4I1 can inhibit CD8^+^ T-cell proliferation and IFN-γ production by Th1 cells,^61^ as well as the ability of driving the differentiation of naive T cells into inducible regulatory T (iTreg) cells.^62^ It is widely acknowledged that B cells, DCs, and macrophages express and produce IL4I1,^63^ whereas its production by MSCs remains unreported.

It is worth noting that while there is a positive correlation between the CXCL10 pathway and the renal disease activity of pediatric SLE,^64^ the relationship between these pathways (IFN-γ−CXCL10 pathway and TNF-α−IL4I1 pathway) and the efficacy of MSC therapy remains unclear. So, an exploratory clinical trial was conducted by our team. A significant improvement in clinical symptoms, such as SLEDAI-2K Score and proteinuria, was observed in five out of the seven patients with LN following hUC-MSC treatment. The correlation between the changes in serum levels of CXCL10 and TNF-α following treatment and LN symptoms is positive. However, the serum concentrations of IFN-γ and IL4I1 in patients are below the detectable limit and cannot be quantified. Thus, it is hypothesized that the alterations in serum CXCL10 may partially indicate the migration of MSCs to the kidneys and their subsequent influence on the renal IFN-γ pathway. Previous studies have indicated that both serum TNF-α concentration and soluble TNF-R1 levels are correlated with disease activity in LN.^65^ However, additional research is required to determine whether changes in serum levels of CXCL10 and TNF-α can serve as biomarkers for the stratification of the disease population and evaluating the effectiveness of MSCs.

In conclusion, this study provides valuable insights into the significant involvement of the CXCL10-CXCR3 axis in the migration of hUC-MSC to the LN kidneys and the subsequent remodeling of the immune microenvironment by MSC-derived IL4I1, which may contribute to the development of new MSC products with enhanced renal homing and renal immune regulation.

## Materials and methods

### hUC-MSCs preparation and dosage

hUC-MSCs were injected intravenously into mice with the product code ‘RY_SW01 Cell Injection’ manufactured by Jiangsu Renocell Biotech Co., Ltd. (Nanjing, China). The acquiescence to clinical trials of lupus nephritis treatment has been granted by the National Medical Products Administration of China (CXSL 2200303), and Phase I and Phase II clinical trials are underway. The hUC-MSC product was recognized based on the minimal criteria recommended by the International Society for Cellular Therapy (ISCT).^66^ And the hUC-MSC product has received certification from the National Institutes for Food and Drug Control of China. Product properties are cell suspension and cell activity is stable for 48 h at 2–8 °C. Mice were given 200 µl hUC-MSCs (8 × 10^5^ cells for each mouse) through the tail vein.

### Mice

Female MRL/lpr and MRL-MpJ mice aged 6–8 weeks were purchased from Shanghai SLAC Laboratory Animal Co. (Shanghai, China). Female Balb/c mice aged 6–8 weeks were purchased from Beijing Vital River Laboratory Animal Technology Co. (Beijing, China). Following the Animal Care and Use Committee guidelines, the mice were housed in individually ventilated cages and maintained under specific pathogen-free (SPF) conditions. The temperature of the feeding environment is between 18 and 29 °C and the relative humidity is maintained at 40–70%. The drinking water was high-pressure sterile water and the feed was autoclaved. The light time was set at 8:00–20:00 to regulate the biorhythms of the mice. All animal experiments were approved by Animal Ethics Committee of China Pharmaceutical University (Animal Authorization Reference Number: 2021-06-023).

Every week MRL/Lpr mice were monitored by urinary protein, urinary creatinine, serum anti-dsDNA antibody level and hair skin defect scores. When the urine protein/creatinine ratio displayed continuous increasing accompanied by a certain degree of hair loss,^13,67^ the mice were screened into the model group which were considered that they had progressed to LN (About 16 weeks).

### MSCs therapy for LN patients and human sample collection

Five active LN patients were recruited from the Affiliated Drum Tower Hospital of Nanjing University Medical School. All the patients aged 22–49 years were diagnosed as LN with a SLEDAI-2K Score of more than 8 and renal biopsy of WHO class III, IV, V, III + V, or IV + V. All the patients were treated with allogenic hUC-MSCs transplantation (2 × 10^6^ cells/kg) and provided informed consent for the collection of peripheral blood. In order to further exclude the influence of immunosuppressants on the action of MSCs, the immunosuppressants were withdrawn 1 week before MSC infusion. The clinical details of the patients are shown in Table 1. Blood samples of the control group were derived from healthy people, and kidney samples of the control group were derived from paraneoplastic tissue in patients with renal cancer. This study was approved by the Ethics Committee of The Affiliated Drum Tower Hospital of Nanjing University Medical School (No.SC-2017-002) and registered on ClinicalTrials.gov (Identifier: NCT01741857).

### Detection method of hUC-MSC in mouse tissues

We have developed a sensitive, specific, and reliable Q-PCR method based on the Alu gene. Quantitative polymerase chain reaction (PCR) primers were created to target the unique human-specific sequence of Alu, focusing on the primer set for human-specific Alu repeats.^66^

hUC-MSCs (40000, 20000, 10000, 5000, 2500, 1250, 625, 312.5, 156.25 cells) were mixed with 5 mg mice organ tissues to extract gDNA. Total genomic DNA from mouse tissue was extracted with the FastPure Cell/Tissue DNA Isolation Mini Kit (Vazyme, China) in accordance with the manufacturer’s protocol. Q-PCR assay of gDNA was performed with SYBR Green Mix (Bio-Rad, California, USA). The standard curve of cycle threshold values (Ct) plotted against cell number (in logarithmic form) was generated through qPCR amplification. Each test tissues were assayed together with the corresponding standard curves, and the number of hUC-MSCs per unit weight in test tissues was calculated according to the standard curve. The accuracy and precision of this method have been validated for preclinical evaluation (data not shown).

### Flow cytometry

Fresh kidneys were finely chopped and subsequently digested at 37 °C Celsius into a single-cell suspension. For single-cell suspensions of the spleen and lymph nodes, organs were minced, and ground using a 70-µm nylon mesh, then washed with isotonic PBS buffer. Following erythrocyte lysis using erythrocyte lysate (Beyotime), the cells were washed multiple times with PBS buffer and then resuspended in FACS containing 2% FBS.

First, before antibody incubation, nonspecific staining was blocked using an anti-mouse CD16/32 antibody (BioLegend). Second, stained with CD45-APC/fire 750, CD4-FITC, and CXCR3-PE in a dark environment at 4 °C. Third, samples were punched and fixed using the Transcription Factor Buffer Set (BD Pharmingen) at 4 °C. Fourth, stained with T-bet-PE/Cy7, ROR-PE-610 in a dark environment at 4 °C. Finally, suspension with FACS containing 2% FBS.

For the flow assay, Th1 cells were co-cultured with hUC-MSCs. Th1 cells were stimulated by adding a cell stimulation cocktail 6 h before collection of Th1 cells. Staining with CD4-BV605, IFN-γ-PE. Finally, suspension with FACS containing 2% FBS.

The stained cells were analyzed using a CytoFLEX S multicolor flow cytometer and processed with FlowJo software (BD Biosciences).

### Splenic T cell cultures and induced differentiation to generate Th1 cells

Add PBS containing 2 µg/mL anti-CD3 antibody (Biolegend, 100339) to the 48-well plate and incubate at 37 °C for 2 h. C57 mice were euthanized, the spleen was removed in a sterile environment, placed on a 70 µm sterile sieve and the spleen was ground to pass through the sieve. Centrifuge at 350× *g*, 4 °C, and discard the supernatant. Following erythrocyte lysis using erythrocyte lysate (Beyotime), the cells were washed multiple times with PBS buffer. Add a biotin-antibody cocktail (Miltenyi) and anti-biotin microbeads and incubate at 4 °C for 30 min. Naive T cells were enriched by magnetic selection (Miltenyi) and positive cell suspensions were collected and added to 48-well plates incubated in advance to induce differentiation by adding a differentiation medium. Induced differentiation medium contains IL-12 (20 ng/mL, Peprotech), IL-2 (20 ng/mL, peprotech), anti-IL4 (10 µg/mL, Peprotech), anti-CD28 (5 µg/mL, Peprotech).

### Single-cell RNA sequencing analysis

Mouse kidneys were initially processed for glomerular extraction via enzymatic digestion.^68^ In brief, minced kidneys were subjected to digestion with collagenase V (1 mg/mL in TESCA buffer), followed by gradient centrifugation and differential adhesion techniques to predominantly isolate glomeruli. The isolated glomeruli were then dissociated into single cells.^69^ Approximately 8000 cells were captured and subjected to barcoding of released RNA through reverse transcription within individual Gel Bead-In-Emulsions (GEMs). Reverse transcription was conducted using an S1000TM Touch Thermal Cycler (Bio-Rad) at 53 °C for 45 min, followed by 85 °C for 5 min, and then held at 4 °C. The resulting cDNA was amplified, and its quality was evaluated using an Agilent 4200, with procedures executed by CapitalBio Technology, Beijing.

Raw sequencing data were processed using the Cell Ranger software (10x Genomics, v1.1.0) for FASTQ file generation and alignment. For data filtration and unsupervised clustering, the gene expression matrix from Cell Ranger was utilized for downstream analysis in R (v4.2.1). Normalization, dimensionality reduction, and cell clustering were conducted using Seurat (R package, v4.2.0). The selection criteria for cells were established based on prior studies, considering the median gene count and mitochondrial gene percentage in samples. Cells with fewer than 200 or more than 3000 genes (indicative of potential doublets), a mitochondrial gene percentage exceeding 10%, or a ribosomal gene percentage over 5% were excluded. In addition, DoubletFinder (v 2.0.3) was employed to identify and eliminate doublets within each dataset.

Dimensionality reduction was achieved through Principal Component Analysis (PCA), where the first 10 principal components were selected for generating clusters. These clusters were identified using both the K-means algorithm and a graph-based approach. In addition, CellChat (v1.6.1) was employed to examine the differences in intercellular communication among various cell types. This step was crucial for understanding the complex interactions within the cellular milieu.

The differential gene expression within each cluster was analyzed using DESeq2 (v1.38.3). This analysis played a key role in identifying genes with unique or significantly varied expression across clusters. Based on the expression levels of Cxcl10, cells were categorized into HIGH and LOW expression groups. The expression profiles of these marker genes in each cluster were visualized using the ggplot2 package (v3.3.6). Furthermore, to understand the biological implications of these expression patterns, Gene Ontology (GO) enrichment analysis and Kyoto Encyclopedia of Genes and Genomes (KEGG) pathway enrichment analysis was performed using Cluster Profiler (v4.6.2). These analyses focused on differentially expressed RNAs, especially those with a fold change greater than 2, providing valuable insights into the molecular underpinnings of the observed phenomena.

### Database data analysis

Figure 2a data from ‘ERCB Lupus TubInt Dataset Summary’, Data demonstrate top 23 genes most altered in lupus nephritis patients relative to healthy controls. Figure S3c from the GEO database (GSE149050), data demonstrating the relevant cytokine signaling genes that are elevated in the PBMC of lupus nephritis patients with high blood levels of IFN-γ relative to those with low levels of IFN-γ.^70^ 126 differential genes including CXCL10 were obtained.

### Statistical analysis

Statistical analyses were performed using GraphPad Prism 8 software (GraphPad Software Inc, San Diego, CA). To assess statistical significance, the Student’s *t* test was employed to compare two groups. For comparisons involving more than two groups, two-way analysis of variance (ANOVA) with Dunnett’s test or one-way ANOVA with Tukey’s test was utilized. Data are described by mean values ± standard deviation (SD), unless otherwise specified. *P* < 0.05 was considered statistically significant.

## Supplementary information

Supplementary Materials for Renal remodeling by CXCL10-CXCR3 axis-recruited mesenchymal stem cells and subsequent IL4I1 secretion in lupus nephritis

Supplementary material for WB and transwell

Study Protocol

## Data Availability

The array data from lupus nephritis patients and healthy controls is sourced from ERCB Lupus TubInt Dataset Summary (not yet published). The PBMC data of lupus nephritis patients is from the GEO database. The GEO accession number is GSE149050. The scRNA-seq raw data generated in this study have been deposited at NCBI and GEO database (PRJNA1170255, GSE279823). All other data and code analyzed during this study are available from the corresponding author upon reasonable request.
