# Supplementary material for: Renal remodeling by CXCL10-CXCR3 axis-recruited mesenchymal stem cells and subsequent IL4I1 secretion in lupus nephritis
Source: Signal Transduct Target Ther. 2024 Nov 18;9:325. doi: 10.1038/s41392-024-02018-5 (PMC11574084; doi:10.1038/s41392-024-02018-5)
Supplement: Supplementary file 1 — Supplementary Materials for Renal remodeling by CXCL10-CXCR3 axis-recruited mesenchymal stem cells and subsequent IL4I1 secretion in lupus nephritis [file 41392_2024_2018_MOESM1_ESM.docx]

Supplementary Materials for

Renal remodeling by CXCL10-CXCR3 axis-recruited mesenchymal stem cells and subsequent IL4I1 secretion in lupus nephritis

Qixiang Zhang^1†^, Yunlong Shan^1†,^*, Luping Shen^1, 4†^, Qi Ni^1†^, Dandan Wang^2^, Xin Wen^2^, Huanke Xu^1^, Xiaoyan Liu^1^, Zhu Zeng^1^, Jingwen Yang^1^, Yukai Wang^1^, Jiali Liu^1^, Yueyan Su^3^, Ning Wei^1, 3^, Jing Wang^3^, Lingyun Sun^2^*, Guangji Wang^1^*, Fang Zhou^1^*

Correspondence to: [zf1113@163.com](mailto:zf1113@163.com) (to F.Z.) or [guangjiwang@hotmail.com](mailto:guangjiwang@hotmail.com) (to G.W.), [lingyunsun@nju.edu.cn](mailto:lingyunsun@nju.edu.cn) (to L.S.), [immunometabolism@163.com](mailto:immunometabolism@163.com) (Y.S.)

**This PDF file includes:**

Materials and Methods

Figures S1 to S8

Tables S1 to S2

### Materials and Methods

#### *Imaging of RFP-MSCs in mice vivo*

hUC-MSCs were transfected in vitro with Lentivirus (Jiman Biotechnology, China) for stable expression of RFP fluorescent protein. Mice were given 200 µl RFP-hUC-MSCs (8 × 10^5^ cells/mice) through the tail vein. At the indicated time points (6 hours, 1 day, 3 days), mice were anesthetized with isoflurane and sacrificed; anticoagulated blood and selected organs were collected. Organs were washed by PBS, and fluorescence intensities were measured by an IVIS (Caliper Life Sciences, Hopkinton, MA) with filter set as follows: Excitation/Emission 594/647 nm.

#### *Cell culture and reagents*

HUVEC cells, a human umbilical vein endothelial cell line, were purchased from Zhongqiao Xinzhou (Shanghai, China). HRGEC Cells, a human glomerular endothelial cell line, were purchased from Procell Life Science and Technology Co. (Wuhan, China). HRMC Cells, a human renal mesangial cell line, were purchased from Procell Life Science and Technology Co. (Wuhan, China). HK2 Cells, a human renal cortex proximal tubule epithelial cell line, were purchased from Zhongqiao Xinzhou (Shanghai, China). RAW264.7 cells were purchased from Zhongqiao Xinzhou (Shanghai, China), which were cultured in DMEM medium containing 10% inactivated serum, and trypsin digestion of cells was prohibited. All cells were cultured at 37°C in a humidified incubator containing 5% CO2.

#### *mRNA Extraction and qRT‐PCR Amplification*

Total mRNA was extracted with RNAiso Plus (Takara, Japan) based on the manufacturer’s protocol, the concentration and purity were detected by the Colibri Spectrophotometer (Berger, Germany). Then, total mRNA was converted to complementary DNA by HiScript III RT SuperMix (Vazyme, China).

The qPCR assay of cDNA was performed in a volume of 15 µl that contained 7.5 µl qPCR SYBR Green Mix (Bio-Rad, California, USA), 1 µl forward and reverse primers, and 200 ng template cDNA diluted in water. The Bio-Rad C1000 server system (Bio-Rad, California, USA) was used to determine mRNA levels. The 2^−ΔΔCt^ method was utilized for quantitative analysis. The mRNA levels were calculated with Beta‐actin (ACTB) or GAPDH as reference genes.

All the primers were synthesized by Sangon Biotech (Shanghai) Co., Ltd. (Shanghai, China). Table S1 shows primer sequences.

#### *Western blotting analysis*

Proteins were extracted in RIPA buffer supplemented with EDTA-free protease inhibitor cocktail (Roche Diagnostics) and phosphatase inhibitor cocktail 3 (Sigma). Proteins were run on 10% gradient gel (YaMei) and blotted onto a nitrocellulose membrane. Antibodies for immunoblotting were as follows: CXCR3 (monoclonal rabbit, Abcam), CXCR4 (monoclonal rabbit, Abcam), CXCR5 (monoclonal rabbit, Abcam), GAPDH (monoclonal rabbit, CST), IRF1 (monoclonal rabbit, Abcam), KPNA4 (monoclonal rabbit, Abcam), Tubulin (monoclonal rabbit, Proteintech), Lamin B1 (monoclonal rabbit, CST). Images were captured and analyzed on a Bio-Rad Chemiluminescent Imaging System. All uncropped scans of western blotting are presented in the Source Data file.

#### *Cell transfection*

Knockdown of *CXCR3, IFNGR, IRF1,* and *KPNA4* in hUC-MSCs which for in-vitro experiment were used by small interfering RNA (siRNA), respectively. SiRNA were synthesized by Jiman Biotechnology (Shanghai, China). The oligodeoxynucleotide sequences used in this study are presented in Table S2. Lipofectamine RNAiMAX transfection reagent (Invitrogen, USA) was used for transfection according to the manufacturer's protocol. Transfection efficiency was measured by qRT‐PCR or western blotting analysis.

*CXCR3* knockdown, *CXCR3* overexpression and *IL4I1* knockdown in hUC-MSCs which for in-vivo experiment were used by lentiviruses (Jiman Biotechnology, China), respectively. Lentivirus were added to complete medium with a multiplicity of infection (MOI) of 5, then Polybrene was added to a final concentration of 8 µg/mL and gently mixed. Each of the above components was added in sequence. After 48 hours of incubation at 37°C, the lentivirus-containing medium was aspirated and replaced with fresh medium, and the cells were collected for subsequent experiments after 24-36 hours of further incubation. Transfection efficiency was examined by inverted fluorescence microscopy or qRT‐PCR.

#### *Enzyme‐linked immunosorbent assay (ELISA)*

Enzyme-linked immunosorbent assay (ELISA) kits for protein level were as follows: Mouse Cxcl9, Cxcl10, Cxcl12, Cxcl13, IFN-γ, TNF-α are all from Wuhan Huamei (Wuhan, China), Human CXCL9, CXCL10 are both from Wuhan Huamei (Wuhan, China), Mouse anti-dsDNA antibody is from FUJIFILM Wako Shibayagi (Japan). All experiments were conducted according to the manufacturer's instructions.

#### *Transwell migration assay*

hUC-MSCs (WT or *CXCR3* knock out) were seeded in the upper chamber of an 8-μm pore size transwell 24-well plate (Corning Incorporated, Corning, ME, USA). Medium supplemented with human chemokine CXCL10 (R&D) or MRL/lpr kidney homogenate or MRL/MpJ kidney homogenate, or anti-CXCL10 antibody (R&D) or medium supernatant collected after HUVECs stimulation by IFN-γ was filled in the lower chamber. The upper chamber cells were stained with crystalline violet, imaged using microscopy, and counted using Image J.

#### *Co-Immunoprecipitation (Co-IP)*

Total protein was extracted using an appropriate amount of modified RIPA buffer (with a protease inhibitor). A small amount of supernatant was used as input for western blotting analysis and the remaining supernatant was incubated overnight at 4°C with the relevant antibody. After overnight incubation, add the appropriate amount of protein beads (Millipore) and incubate at 4°C for 2-4 h. Then place the EP tube on a magnetic adsorption rack, discard the supernatant and wash 3-4 times with 1 mL of PBS buffer. Add 30 μl of 1 × SDS loading buffer and cook at 100°C for 5 minutes. Analysis by western blotting.

#### *In situ hybridization*

To detect fluorescent *Cxcl10* mRNA expression in MRL/lpr and MRL/MpJ mouse glomeruli, we performed an in-situ hybridization procedure. The mouse Cxcl10 monoclonal factor probe used for in situ hybridisation was synthesised by Bio-Techne (Nasdaq, USA). Supporting reagents were provided by Bio-Techne (Nasdaq, USA). Frozen kidney tissue sections after fixation were assayed in combination according to the manufacturer's instructions. Images were then viewed with an Olympus Fluoview FV3000 confocal microscope (Olympus, Tokyo, Japan).

#### *Immunofluorescence*

Tissues were fixed in 4% paraformaldehyde for 24 hours and then sequentially dehydrated overnight with 20% sucrose and 30% sucrose in PBS. Tissue or cells are blocked at room temperature with 0.1% Triton X-100 in PBS and 5% donkey serum for 1 h. Tissue slides were incubated overnight at 4°C with diluted FITC anti-mouse IgG (1:100, BioLegend), then washed with PBS, and finally stained with DAPI and mounted.

Cells were cultured in confocal culture dishes (Corning) and fixed in 4% paraformaldehyde for 15 minutes at room temperature. Cells were incubated overnight at 4°C with diluted primary antibodies, IRF1 (1:500, Abcam), KPNA4 (1:500, Abcam). Alexa Fluor 594 anti-rabbit or 488 anti-mouse secondary antibodies were applied, washed with PBS, and stained with DAPI (Beyotime). The images were then observed using an Olympus Fluoview FV3000 confocal microscope (Olympus, Tokyo, Japan).

#### *Detection of protein and creatinine in the urine of mice*

Total urine was collected from mice for 12 hours using mouse metabolite collection cages and stored at -80°C. According to the manufacturer's instructions, the level of total protein in urine was measured with the Pierce BCA Protein Assay Kit (Thermo, Massachusetts, USA). The level of creatinine in urine was measured with the Creatinine (Cr) Colorimetric Assay Kit (Elabscience, Wuhan, China).

#### *Dual‐luciferase reporter assay*

A dual-luciferase reporter assay was used to assess the direct binding between IRF1 and CXCL10 promoter regions. A recombinant plasmid containing a binding sequence or promoter sequence was constructed by Jiman Biotechnology (Shanghai, China). HE293 cells were co-transfected with the IRF1 plasmid or negative control (NC) with the CXCL10 promoter plasmid by Lipofectamine 2000 reagent (Thermo Fisher Scientific). Luciferase activity was detected by the Duo-Luciferase HS Assay Kit (GeneCopoeia, USA) according to the manufacturer's instructions.


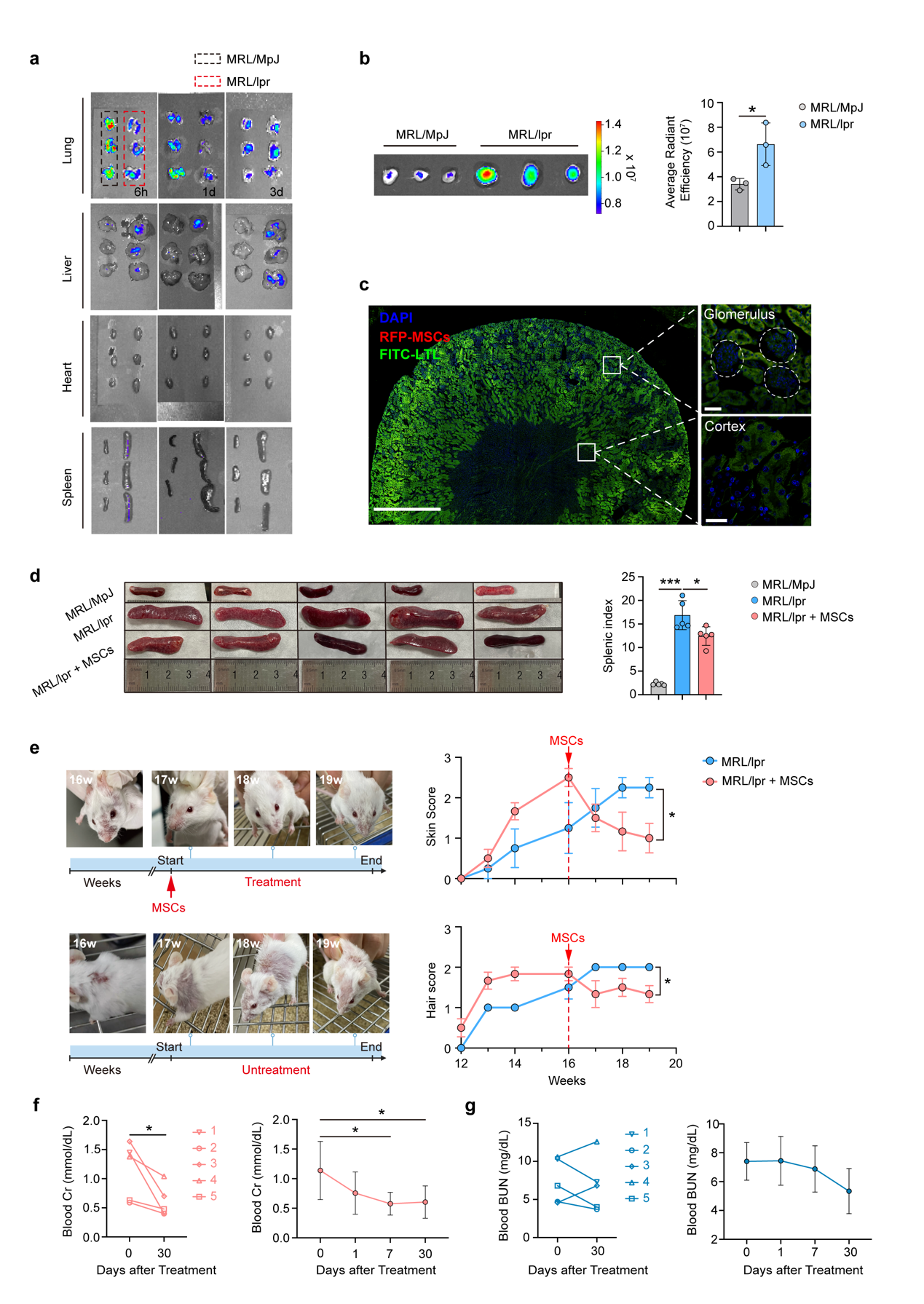


### Figure. S1. Increased recruitment of hUC-MSCs to kidney in MRL/lpr mice alleviates renal of lupus nephritis.

**a** Fluorescence intensity of RFP-MSCs in lung, heart, liver, spleen is determined by IVIS Imaging System (n=3). **b** Fluorescence intensity of lymphatic nodes at 1 day after intravenously administration of RFP-hUC-MSCs is determined by IVIS Imaging System (n=3). **c** Representative images of RFP-MSCs distribution in kidney sections from MRL/MpJ mice. Scale bar: Full scan of kidney section (1000 µm), Glomerulus (50 µm), Cortex (10 µm). **d** Spleen weight of MRL/MpJ mice and MRL/lpr mice at 21 days treated with or without hUC-MSCs (n=5). Representative photographs of the spleen are shown. **e** Skin damage and hair loss in MRL/lpr mice treated with or without hUC-MSCs (n=5). Representative photographs of the appearance of the mice are shown. **f, g** Changes in blood creatinine (f) and blood BUN (g) levels in 5 lupus nephritis patients treated with hUC-MSCs. Data represent mean ± SD. Two-tailed unpaired equal-variance t-test. **P* < 0.05, ****P* < 0.001.

**
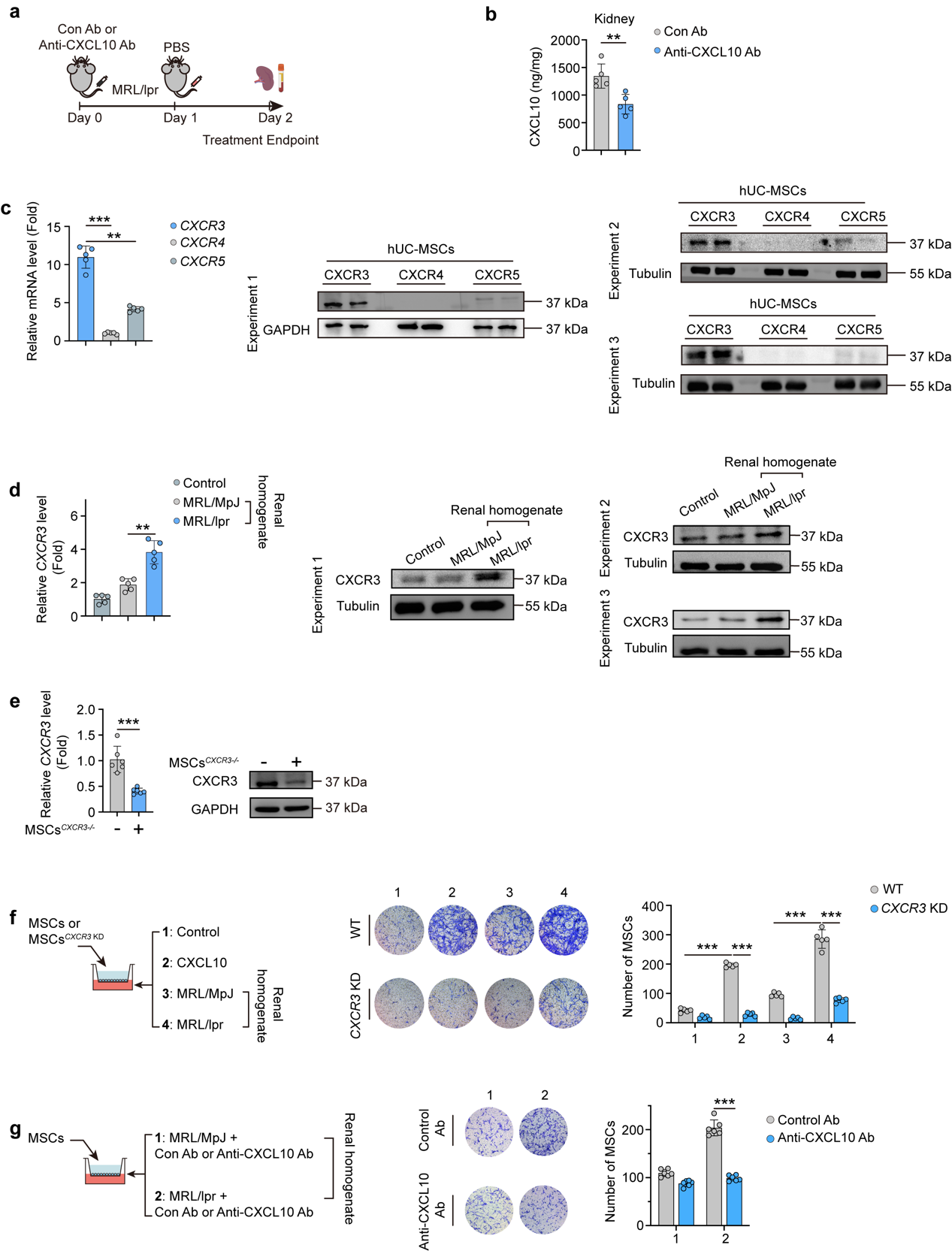
**

### Figure. S2. Disruption of CXCL10-CXCR3 axis suppresses hUC-MSCs recruitment to LN kidney.

**a** Scheme illustrating for kidney Cxcl10 and blood anti-dsDNA antibody levels in MRL/lpr mice pretreated with the antibody for 2 days. Mice were treated with 20 µg of isotype control or anti-CXCL10 antibody. **b** The protein level of Cxcl10 in kidney from MRL/lpr mice after 2 days they were treated with 20 µg isotype control or anti-CXCL10 antibodies (n=5). **c** The mRNA and protein level of chemokines as indicated in hUC-MSCs were detected by Q-PCR (n=5) and western blotting (n=2, three independent experiments), respectively. **d** hUC-MSCs were stimulated with or without MRL/MpJ or MRL/lpr mice renal homogenates for 30 minutes. *Cxcr3* mRNA level (n=5) and Cxcr3 protein level (three independent experiments) of hUC-MSCs were detected. **e** The mRNA expression and protein expression of CXCR3 are respectively measured in hUC-MSCs*^NC^* and hUC-MSCs*^CXCR3 KD^*. hUC-MSCs are transfected with *CXCR3* siRNA or negative control (NC) for 24 h, respectively. Each data point represents an independent experiment. **f, g** Cell migration was assessed by transwell assays. Representative images and numbers of hUC-MSCs*^NC^* (Negative Control) and hUC-MSCs*^CXCR3 KD^* recruited by supernatant with or without CXCL10 (500 ng/mL) or renal homogenate from MRL/lpr and MRL/MpJ mice (f) (n=3). Representative images and numbers of hUC-MSCs recruited by supernatant with MRL/lpr or MRL/MpJ mice renal homogenate in the presence of isotype control or anti-CXCL10 antibody, respectively (g) (n=3). Data represent mean ± SD. ***P* < 0.01, ****P* < 0.001.


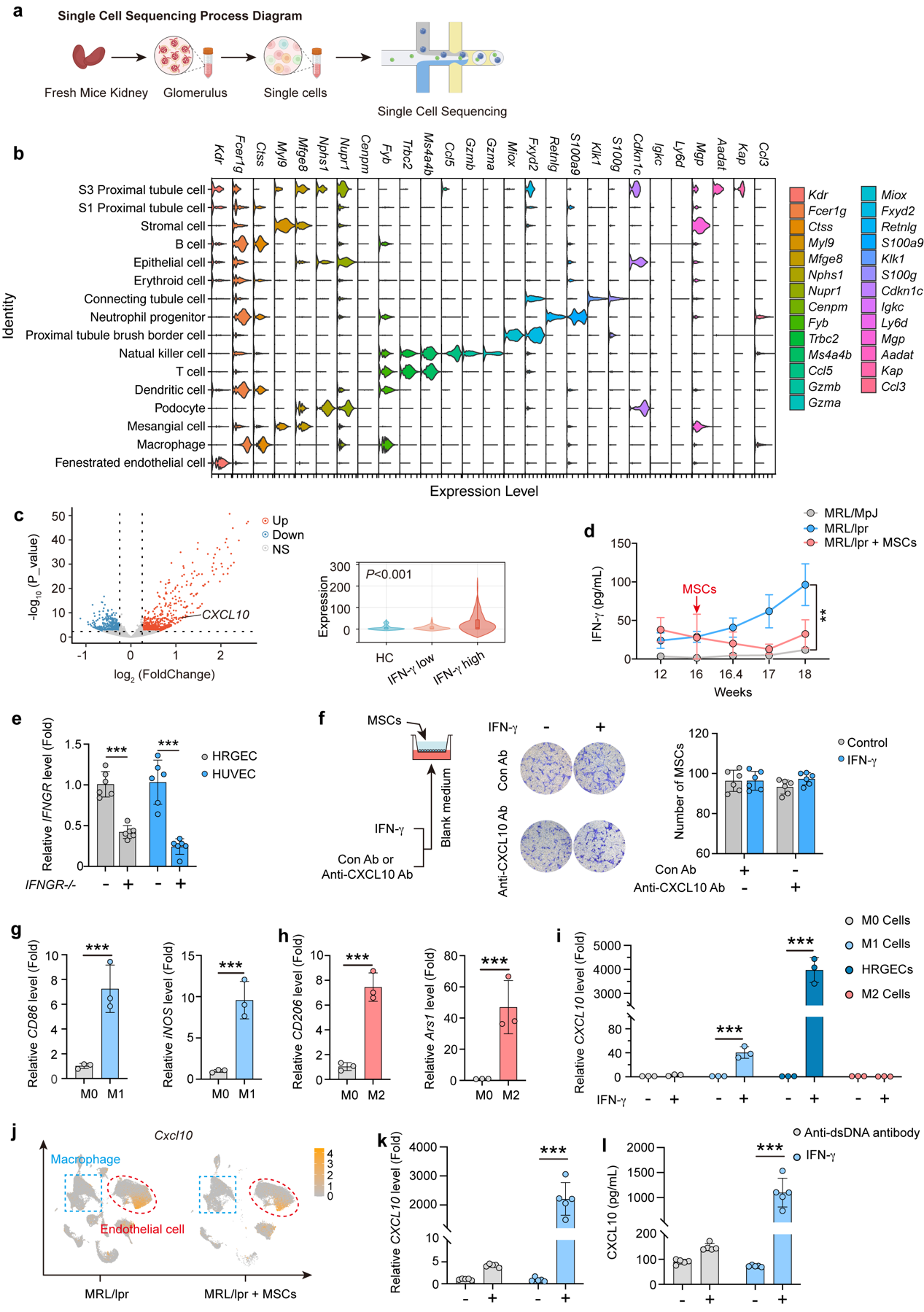


### Figure. S3. Renal CXCL10 is primarily derived from glomerular vascular endothelial cells via IFN-γ/IFN-γ R pathway in LN.

**a** Schematic diagram of the sampling process for single-cell RNA sequencing of MRL/lpr mouse glomeruli. Figure created with BioRender (<https://biorender.com>). **b** Violin plots of marker gene expression in each cluster of glomerulus single cell sequencing. **c** Transcriptome analysis of interferon responsive (IFN-γ high) SLE, interferon non-responsive (IFN-γ low) SLE and healthy control (HC) human PBMC samples in the GEO (GSE149050) database. Each box represents a patient. **d** The concentrations of IFN-γ in blood from MRL/MpJ mice and MRL/lpr mice treated with or without hUC-MSCs (n=5). **e** mRNA expression of *IFNGR* are measured in HRGECs and HUVECs (Negative Control or *IFNGR* knockdown) (n=6). HUVECs are transfected with *IFNGR* siRNA or negative control (NC) for 24 h, respectively. Each data point represents an independent experiment. **f** Representative images and numbers of hUC-MSCs recruited by blank medium which were added IFN-γ (50 ng/mL) and anti-CXCL10 or isotype control antibody (5 µg/mL), (n=3), cell migration was assessed by transwell assays (8 µm). **g** Expression levels of *CD86* and *iNOS* genes, which are characteristically expressed in M1-type macrophages (n=3). M1 macrophages were obtained by inducing RAW264.7 cells with 100 µg/mL LPS for 24 hours. **h** Expression levels of *CD206* and *Ars1* genes, which are characteristically expressed in M2-type macrophages (n=3). M2 macrophages were obtained by inducing RAW264.7 cells with 10 ng/mL IL-4 for 24 hours. **i** Expression levels of *CXCL10* in M0, M1, M2 macrophages, and endothelial cells with or without IFN-γ (100 ng/mL) stimulation for 24 hours (n=3). **j** UMAP plot of *Cxcl10* gene levels in glomerular endothelial cells (red circle) and macrophages (blue box) of MRL/lpr mice at 21 days treated with or without hUC-MSCs. **k, l** The gene level of *CXCL10* in the cells was measured by Q-PCR (k) and the protein level of CXCL10 in the culture medium supernatant was measured by ELISA (l) after stimulating HUVEC for 24 hours with anti-dsDNA antibody (200 ng/mL) and IFN-γ (100 ng/mL), respectively (n=5). Data represent mean ± SD. ***P* < 0.01, ****P* < 0.001.


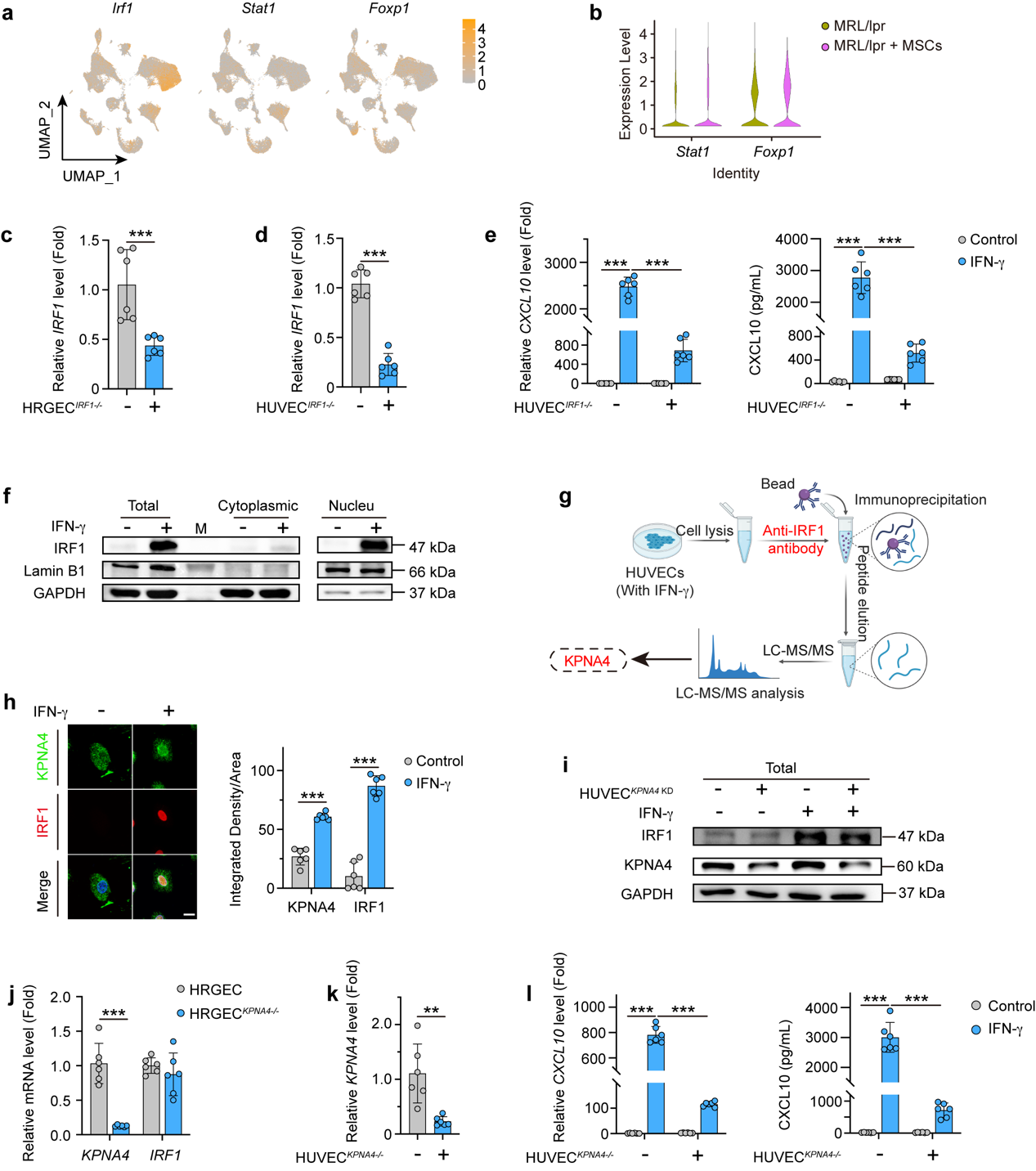


### Figure. S4. Nuclear transport of IRF1-KPNA4 mediates IFN-γ-induced secretion of CXCL10 from endothelial cells.

**a** UMAP plot showing the distribution of *Irf1*, *Stat1* and *Foxp1* expression in glomeruli of MRL/lpr mice. **b** Violin plots of *Stat1* and *Foxp1* gene levels in glomerular endothelial cells of MRL/lpr mice at 21 days treated with or without hUC-MSCs. **c, d** *IRF1* mRNA expression are measured in HRGECs (c) and HUVECs (d) with or without *IRF1* knockdown (n=6). HRGECs and HUVECs are transfected with *IRF1* siRNA or negative control (NC) for 24 h, respectively. Each data point represents an independent experiment. **e** HUVECs*^NC^* and HUVECs *^IRF1 KD^* were stimulated with or without IFN-γ (50 ng/mL) for 12 h. *CXCL10* mRNA level of cells and CXCL10 protein level of medium supernatant were detected (n=6). **f** Total protein, cytoplasmic protein and nucleoprotein expression of IRF1 are analyzed by western blotting in HUVEC cells with or without human IFN-γ (50 ng/mL) for 8 h. Lamin B1 as an internal reference protein for nucleoprotein, GAPDH as an internal reference protein for total and cytoplasmic protein. **g** Lysates of HUVEC cells with or without human IFN-γ (50 ng/mL) for 8h were subjected to immunoprecipitation (IP) using anti-IRF1 antibody and the enriched proteins were identified by LC-MS-MS for their protein composition. The figure created with BioRender (<https://biorender.com>). **h** KPNA4 and IRF1 protein expression are analyzed by immunofluorescence in HUVECs with or without human IFN-γ (50 ng/mL) for 8 h (n=6). Green for KPNA4, red for IRF1, blue for DAPI in the immunofluorescence assay. Scale bar: 20 µm. **i** Total protein expression of IRF1 and KPNA4 are respectively analyzed by western blotting in HUVECs*^NC^* and HUVECs*^KPNA4 KD^* with or without human IFN-γ (50 ng/mL) for 8h. **j** *KPNA4* and *IRF1* mRNA expression are measured in HRGECs*^NC^* and HRGECs*^KPNA4 KD^* (n=6). HRGECs are transfected with *KPNA4* siRNA or negative control (NC) for 24 h, respectively. **k** *KPNA4* mRNA expression are measured in HUVECs*^NC^* and HUVECs*^KPNA4 KD^* (n=6). HUVECs are transfected with *KPNA4* siRNA or negative control (NC) for 24 h, respectively. **l** HUVECs*^NC^* and HUVECs*^KPNA4 KD^* were stimulated with or without IFN-γ (50 ng/mL) for 12 h. The *CXCL10* mRNA level of cells and CXCL10 protein level of medium supernatant were detected (n=6). Data represent mean ± SD. ***P* < 0.01, ****P* < 0.001.

**
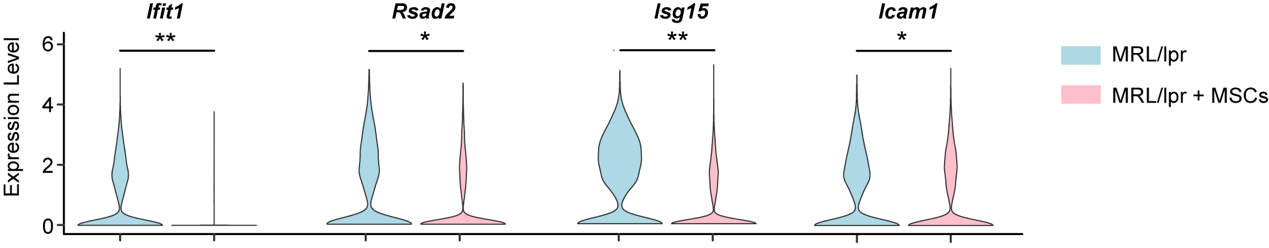
**

### Figure. S5. Amelioration of endothelial inflammation in glomeruli of lupus nephritis by hUC-MSCs.

Violin plots of *Ifit1*, *Rsad2*, *Isg15*, *Icam1* gene levels in glomerular endothelial cells of MRL/lpr mice at 21 days treated with or without hUC-MSCs. Data represent mean ± SD. **P* < 0.05, ***P* < 0.01, ****P* < 0.001.


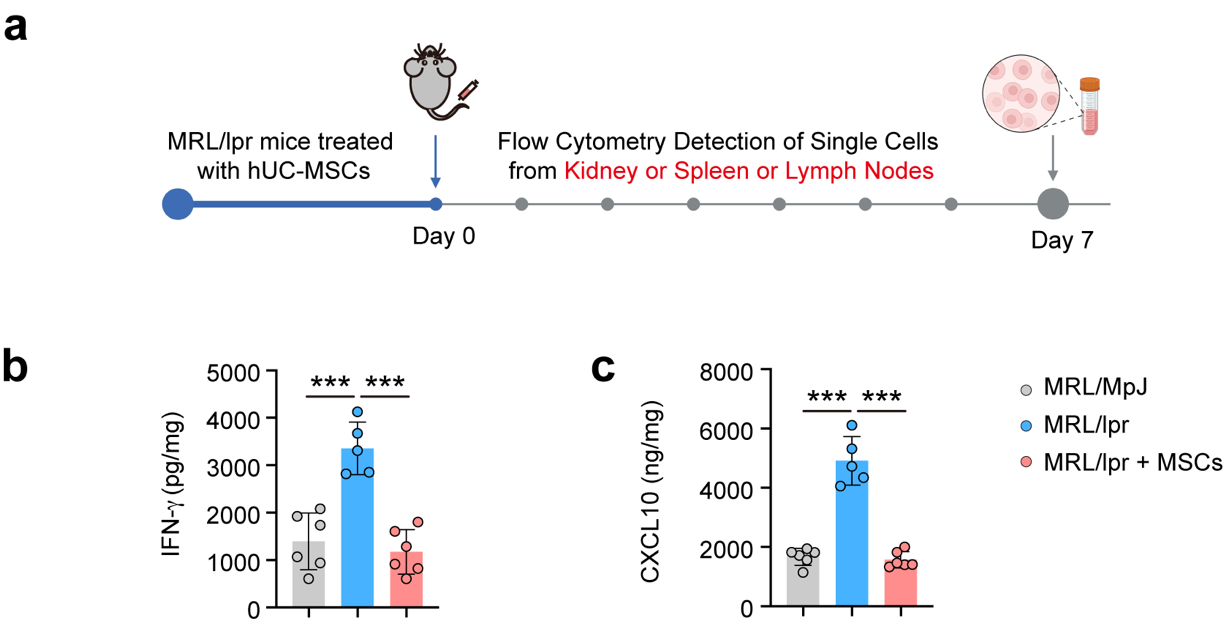


### Figure. S6. hUC-MSCs reduce the infiltration of CXCR3^+^ Th1 cells into lupus nephritis kidney.

**a** Schematic timeline of mice being injected intravenously with hUC-MSCs and single-cell samples detected by flow cytometry. **b, c** IFN-γ (b) and CXCL10 (c) concentrations in kidneys from mice of Fig. 5e, (n=5-6). Data represent mean ± SD. **P* < 0.05, ***P* < 0.01, ****P* < 0.001.


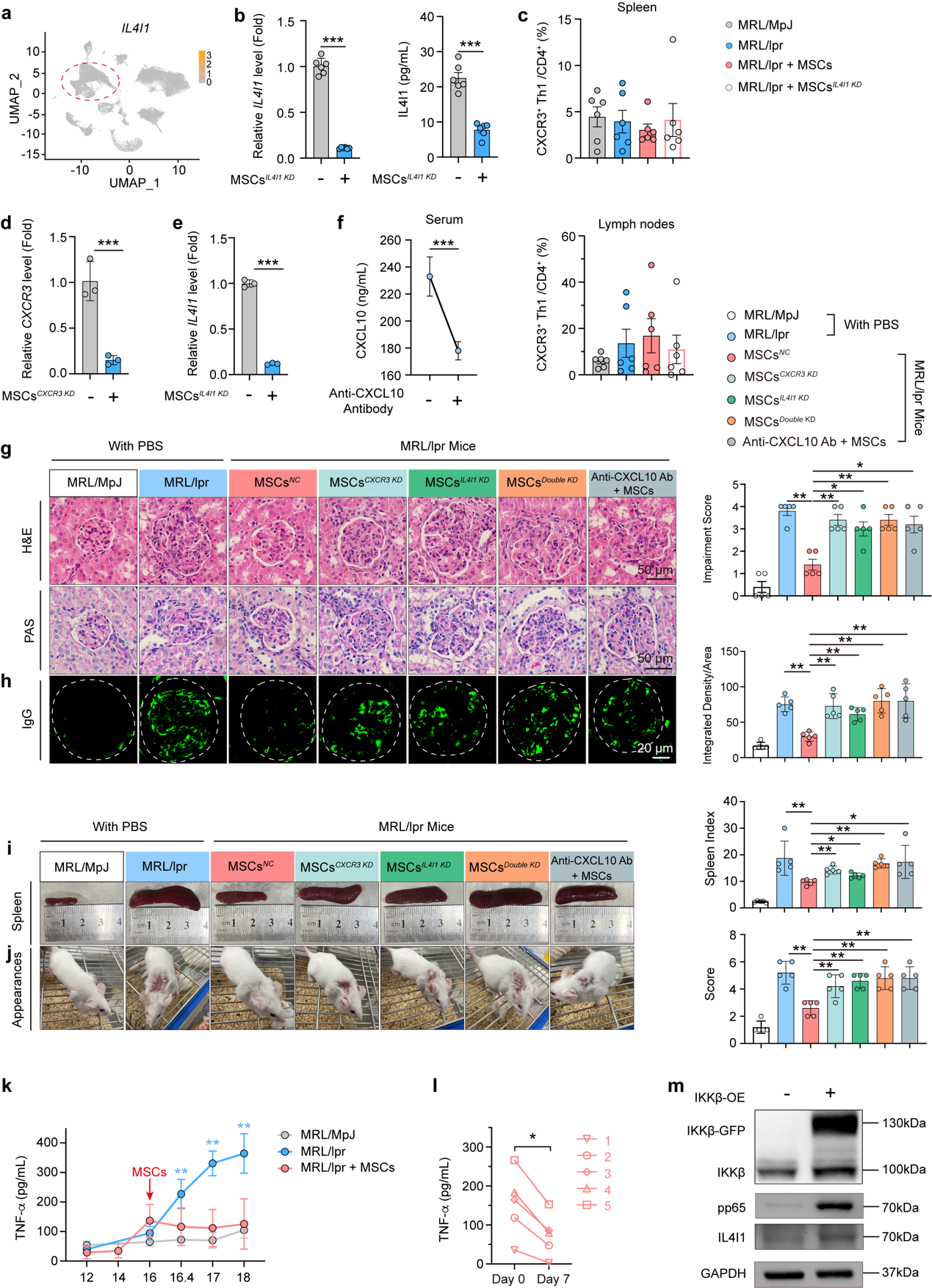


### Figure. S7. IL4I1 derived from hUC-MSCs inhibits renal CXCL10 level and the infiltration of CXCR3^+^ Th1 cell in LN.

**a** UMAP plot of *IL4I1* expression in glomeruli of MRL/lpr mice. **b** The mRNA level and secretory protein of IL4I1 in cells were detected in hUC-MSCs (Negative Control or *IL4I1* knockdown) (n=6). hUC-MSCs are transfected with *IL4I1* lentivirus or negative control (NC) for 48 h, respectively. Each data point represents an independent experiment. **c** Frequencies of CXCR3^+^ Th1**/**CD4^+^ cells in spleen and lymph nodes from MRL/MpJ mice, MRL/lpr mice at 7 days treated with or without hUC-MSCs*^NC^* or hUC-MSCs*^IL4I1 KD^* were detected by flow cytometry (n=6). **d, e** The mRNA levels of *CXCR3* (d) and *IL4I1* (e) in cells were detected in hUC-MSCs (Negative Control or *CXCR3*, *IL4I1* knockdown) (n=3). hUC-MSCs are transfected with CXCR3, IL4I1 lentivirus, or negative control (NC) for 48 h, respectively. Each data point represents an independent experiment. **f** Concentration of CXCL10 in serum from MRL/lpr mice before and after treatment with anti-CXCL10 antibodies (n=5). **g** Glomerular pathological sections of MRL/lpr and MRL/MpJ mice treated with or without hUC-MSCs (n=5). Scale bar: 50 µm. **h** Deposition of immune complexes IgG in glomerulus of MRL/lpr mice at 21 days treated with or without hUC-MSCs (n=5). Scale bar: 20 µm. **i, j** Spleen weight (i) and skin damage (j) of MRL/MpJ mice and MRL/lpr mice at 21 days treated with or without hUC-MSCs (n=5). Representative photographs of the appearance of the mice are shown. **k** TNF-α concentrations in blood from MRL/MpJ mice and MRL/lpr mice treated with or without hUC-MSCs (n=6). **l** Levels of TNF-α in the serum of 5 patients (1-5) with lupus nephritis before and 7 days after treatment with hUC-MSCs. **m** hUC-MSCs were treated with *IL4I1* overexpressing lentivirus for 48 h and then cell lysates were collected, protein expression levels of GFP, IKKβ, p-p65, IL4I1 were respectively measured by western blotting. Data represent mean ± SD. **P* < 0.05, ***P* < 0.01, ****P* < 0.001.


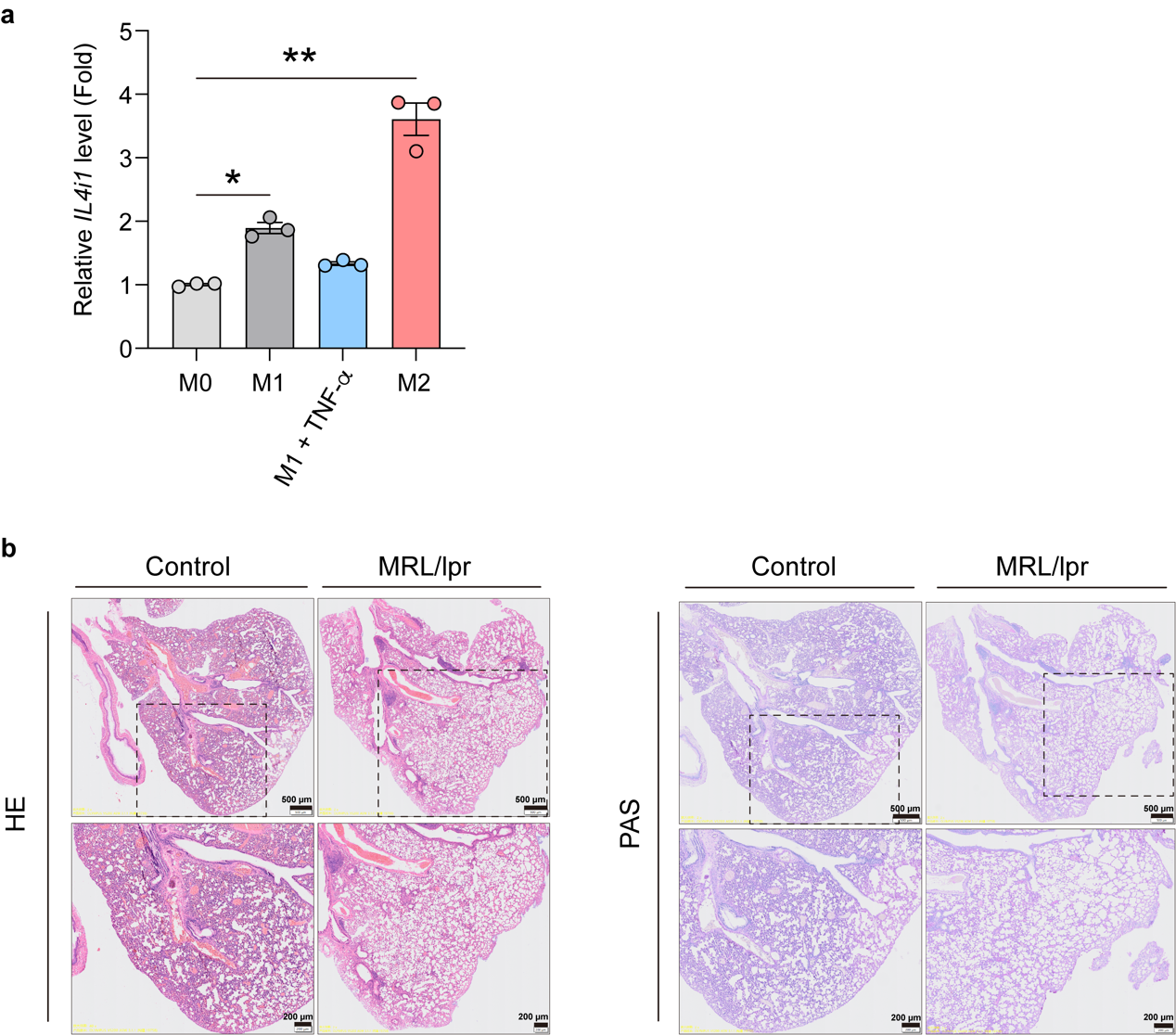


### Figure. S8. a Expression levels of *IL4I1* in M0, M1 and M2 type macrophages (n=3). RAW264.7 cells with 100 µg/mL LPS for 24 hours, and M2 macrophages were obtained by inducing RAW264.7 cells with 10 ng/mL IL-4 for 24 hours. b Histological sections of lungs from both control and MRL/lpr mice were stained using hematoxylin and eosin (H&E) and periodic acid-Schiff (PAS) stains for comprehensive morphological assessment. Scale bar: 500 µm and 200 µm. Data represent mean ± SD. **P* < 0.05, ***P* < 0.01, ****P* < 0.001.

### Table S1.

**Primers for real-time polymerase chain reaction**

| **Gene Name** | **Forward (5’-3’)** | **Reverse (5’-3’)** |
| --- | --- | --- |
| *Actin_Homo* | GAAGGAAGGCTGGAAGAG | GCGTGACATTAAGGAGAAG |
| *Actin_Mus* | CTCCATCCTGGCCTCGCTGT | AACAGTCCGCCTAGAAGCAC |
| *Cxcl15_Mus* | CATTGCCGGTGGAAATTCCTT | TCGAGACCATTTACTGCAACAG |
| *Cxcl5_Mus* | TTGCGGCTATGACTGAGGAAG | TCCAGCTCGCCATTCATGC |
| *IL4I1_Homo* | CCGATCCTGTTATCTGCCTCC | GCCAAGACCCCTTCGAGAAAT |
| *Cxcl9_Mus* | AGTCTTGAAAGCCCATGTGAAA | CCCTCTCCTTCCTCATTCTTACA |
| *Ccl4_Mus* | CTGTCTGCCTCTTTTGGTCAG | TTCCTGCTGTTTCTCTTACACCT |
| *Ccl2_Mus* | TGGAATCCTGAACCCACTTCT | CAGCCAGATGCAATCAATGCC |
| *Cxcl10_Mus* | GGCTCGCAGGGATGATTTCAA | CCAAGTGCTGCCGTCATTTTC |
| *Cxcl13_Mus* | GGGCGTAACTTGAATCCGATCTA | GGCCACGGTATTCTGGAAGC |
| *GAPDH_Homo* | CATGTAGGCCATGAGGTCCACCAC | TGAAGGTCGGTGTCAACGGATTTGGC |
| *Cxcl12_Mus* | TTCTTCAGCCGTGCAACAATC | TGCATCAGTGACGGTAAACCA |
| *IFNGR1_Homo* | TTCCATCTCGGCATACAGCAA | TCTTTGGGTCAGAGTTAAAGCCA |
| *Cxcl9_Mus* | TTTGTAGTGGATCGTGCCTCG | TCCTTTTGGGCATCATCTTCC |
| *KPNA4_Homo* | GCACAGCATTGGACTGAACTA | TCCAGTGATCGAAATCCACCA |
| *IRF1_Homo* | GTCCTCAGGTAATTTCCCTTCCT | GCAGCTACACAGTTCCAGG |
| *CXCL10_Homo* | TGATGGCCTTCGATTCTGGATT | GTGGCATTCAAGGAGTACCTC |
| *CD86_Mus* | TCAATGGGACTGCATATCTGCC | GCCAAAATACTACCAGCTCACT |
| *iNOS_Mus* | GTTCTCAGCCCAACAATACAAGA | GTGGACGGGTCGATGTCAC |
| *CD206_Mus* | CTCTGTTCAGCTATTGGACGC | TGGCACTCCCAAACATAATTTGA |
| *Ars1_Mus* | CTCCAAGCCAAAGTCCTTAGAG | GGAGCTGTCATTAGGGACATCA |

### Table S2.

**siRNA sequences**

| **Target** | **Forward Sequence (5’-3’)** | **Reverse Sequence (5’-3’)** |
| --- | --- | --- |
| *CXCR3*_Homo | GUGCUAAAUGACGCCGAGG | CCUCGGCGUCAUUUAGCAC |
| *IFNGR*_Homo | CACCAACUAAUGUUACAAU | AUUGUAACAUUAGUUGGUG |
| *IRF1*_Homo | GCUCAGCUGUGCGAGUGUA | UACACUCGCACAGCUGAGC |
| *KPNA4*_Homo | CAUUGUUACUGGAACUGAU | AUCAGUUCCAGUAACAAUG |
