# Supplementary figures and images for: Renal remodeling by CXCL10-CXCR3 axis-recruited mesenchymal stem cells and subsequent IL4I1 secretion in lupus nephritis
Source: Signal Transduct Target Ther. 2024 Nov 18;9:325. doi: 10.1038/s41392-024-02018-5 (PMC11574084; doi:10.1038/s41392-024-02018-5)

## Full western blots


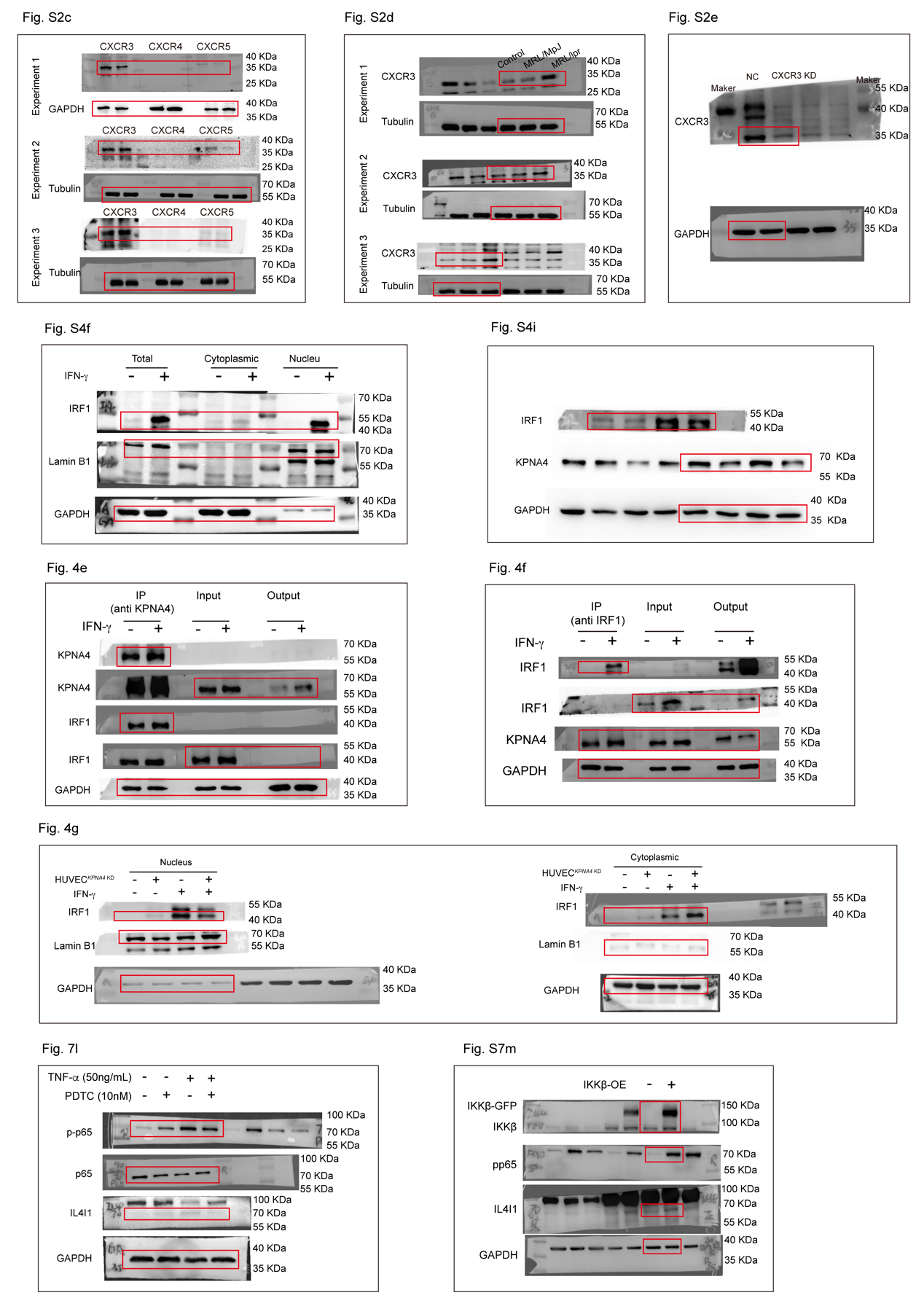


## Transwell complete visual images


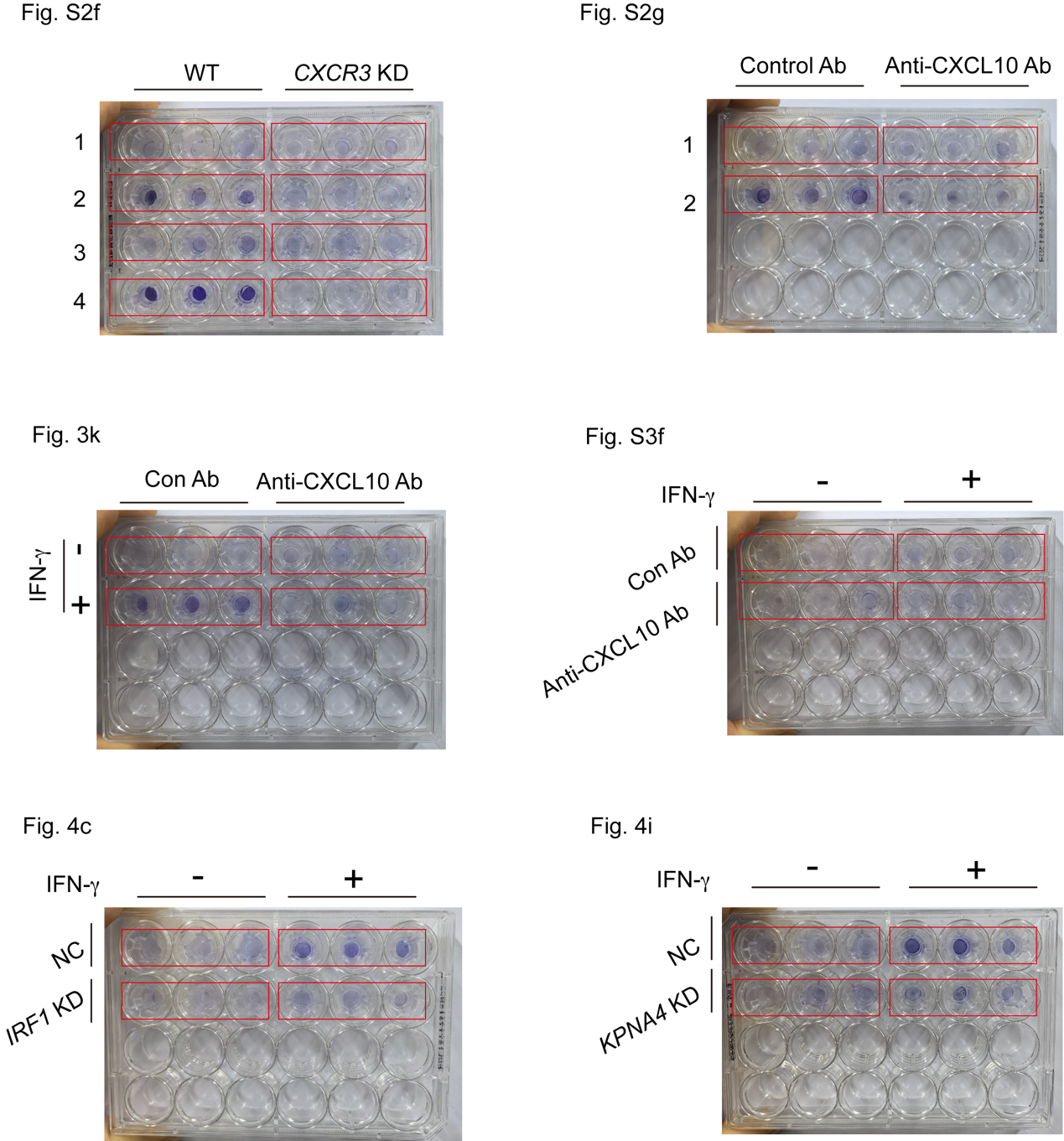

Supplement: Supplementary file 2 — Supplementary material for WB and transwell [file 41392_2024_2018_MOESM2_ESM.docx]
