# Supplementary material for: Renal remodeling by CXCL10-CXCR3 axis-recruited mesenchymal stem cells and subsequent IL4I1 secretion in lupus nephritis
Source: Signal Transduct Target Ther. 2024 Nov 18;9:325. doi: 10.1038/s41392-024-02018-5 (PMC11574084; doi:10.1038/s41392-024-02018-5)
Supplement: Supplementary file 3 — Study Protocol [file 41392_2024_2018_MOESM3_ESM.docx]

# Umbilical cord mesenchymal stem (stromal) cells to treat lupus nephritis: protocol for a prospective clinical trial

## Study design

This is a proposed protocol for a prospective clinical trial, developed in accordance with the Standard Protocol Items: Recommendations for Interventional Trials (SPIRIT) checklist. The Affiliated Drum Tower Hospital of Nanjing University Medical School (Nanjing, China) acts as the initiating sponsor and coordinator of this trial. The observed group will receive intravenous infusion of Umbilical cord mesenchymal stem cells (UC-MSCs).

## Subjects Eligibility

Recruitment methodologies include advertisements in academic media as well as liaisons with general practitioners, rheumatologist.

Inclusion criteria are as follows:

(1) Patients with a documented history of fulfilling at least 4 out of the 11 ACR 1997 SLE classification criteria before randomization; (2) Age between 18 and 65 years at the time of obtaining informed consent; (3) Biopsy-confirmed lupus nephritis with pathological classification of Class III, IV, V, III+V or IV+V (2003 ISN/RPS lupus nephritis histological classification criteria), accompanied by active lesions (activity index ≥ 1 and/or increased chronicity index), within 24 weeks; (4) Urine protein/creatinine ratio > 1.0 or 24-hour urine protein quantification > 1.0 g, with or without active urinary sediment containing red blood cell casts; (5) All subjects receiving concurrent treatment with steroids alone or in combination with antimalarial drugs; (6) Meeting one of the following conditions: a. Never received immunosuppressive agents previously. b. Previously received immunosuppressive agents (including CTX, cyclosporine, TAC, MMF, azathioprine, leflunomide, or sirolimus) but had inadequate response or intolerance to treatment; (7) For women of childbearing potential, a negative serum pregnancy test at screening; (8) Willingness to provide written informed consent and comply with the study protocol requirements and restrictions.

## Exclusion

Exclusion criteria are as follows: (1) Received rituximab or any other B-cell depletion therapy within 24 weeks before screening, or received biologics or small-molecule targeted drugs for immune-related diseases (such as abatacept, infliximab, adalimumab, golimumab, belimumab, tocilizumab, or JAK inhibitors) within 4 weeks before screening, except for herpes zoster vaccine; (2) Received plasma exchange or immunoadsorption therapy within 12 weeks before screening; (3) Prior ineffective or intolerant use of mycophenolate mofetil; (4) Patients with autoimmune diseases other than SLE, including dermatomyositis/polymyositis, mixed connective tissue disease, systemic sclerosis, rheumatoid arthritis, etc., except for patients with secondary Sjögren's syndrome who are allowed to participate in the trial; (5) Blood abnormalities at screening, including any of the following: WBC < 2000/μL, Hb < 6g/dL, PLT < 30000/μL. If any of these abnormalities occur, a repeat screening may be performed, and if the subject meets the inclusion criteria, they can be considered eligible for inclusion; (6) Severe liver or renal impairment at screening, with any of the following abnormalities: total bilirubin > 1.5 times the upper limit of normal (ULN), alkaline phosphatase > 2 ULN, ALT or AST > 2 ULN, eGFR < 30 ml/min or serum creatinine > 265.2 μmol/L. If any of these abnormalities occur, a repeat screening may be performed, and if the subject meets the inclusion criteria, they can be considered eligible for inclusion; (7) Severe uncontrolled cardiovascular disease, neurological disorders, pulmonary diseases (including obstructive lung disease and interstitial lung disease), liver disease, endocrine disorders (including uncontrolled diabetes), or gastrointestinal diseases; (8) Current or recent (within 4 weeks before randomization) severe active or recurrent bacterial, viral, fungal, parasitic, or other infections (including but not limited to tuberculosis and atypical mycobacterial diseases, hepatitis B and C, HIV infection, herpes zoster), or requiring hospitalization and intravenous antibiotic treatment during the screening period; (9) Underwent major surgery within 12 weeks before screening or expected to undergo major surgery during the study period, which is considered to pose unacceptable risks to the patient; (10) Received live vaccines within 12 weeks before randomization or anticipated to receive/require live vaccines during the study period, excluding herpes zoster vaccine; (11) History of malignancies, including solid tumors and hematological malignancies (except for treated and cured); (12) Pregnancy or breastfeeding; (13) If the patients are of reproductive age and do not consent to using effective contraception during the trial.

## Intervention

In this study, the intervention factor is UC-MSCs. The UC-MSCs used in the study will be produced by Jiangsu Renocell Biotech Co., Ltd. that conforms to Good Manufacturing Practice standards. UC-MSCs at passages 4 will be utilized, and the transplantation dose will be 2 × 10^6^ /kg cells based on our preclinical study results. To ensure sterility and viability, UC-MSCs will be suspended in 30mL of sterile normal saline and stored at approximately 4 °C during transportation. The stem cell suspensions will be transported as quickly as possible to enable infusion within 12 hours of dissociation. Throughout the six-month intervention period, all subjects will receive the recommended standard of care concurrently with the assigned interventions.

## Safety indicators and primary and secondary efficacy endpoints

The primary efficacy endpoint of this study is the assessment of the overall response rate (complete response, CR, and partial response, PR) at 24 weeks, defined as a urine protein/creatinine ratio < 0.5, absence of active urinary sediment, and normal serum albumin level. The secondary efficacy endpoints include:

(1) Time to achieve PR and CR in both groups of subjects; (2) Changes in quantified urine protein and/or urine protein/creatinine ratio, serum albumin, serum creatinine, estimated glomerular filtration rate (eGFR), complement levels (C3, C4), autoantibodies (anti-dsDNA, ANA, anti-Smith, anti-RNP, anti-SSA/Ro, anti-SSB/La, anti-phospholipid antibodies) and Coombs test from the screening period to respective follow-up visits; (3) Changes in patient-reported Health Assessment Questionnaire (HAQ) scores and physician's Visual Analog Scale (PhGA-VAS) scores from the screening period to respective follow-up visits.(4) Changes in SLE Disease Activity Index-2000 (SLEDAI-2K), British Isles Lupus Assessment Group-2004 (BILAG-2004) scores, improvement rate in SLE Responder Index from the screening period to respective follow-up visits. (5) Overall response rate (CR + PR) in both groups of subjects at 12 weeks. The safety evaluation includes: (1) Recording of adverse events occurring from the start of subject enrollment until the end of follow-up; (2) Vital sign assessments (blood pressure, pulse, respiratory rate, and temperature) were evaluated every 4 weeks for 24 weeks; (3) Laboratory tests (including complete blood count, urinalysis, liver function, and blood biochemistry) were tested every 4 weeks for 24 weeks.

## Statistical analysis

Descriptive statistics will be used to summarize baseline characteristics, efficacy outcomes, and safety data. For the safety analysis set, all safety indicators will be obtained from those who have undergone UC-MSC. In the full analysis set, subjects will be excluded if they received no intervention or never gave any evaluation outcome. For the baseline characteristics, continuous variables will be expressed as means ± standard deviations, while categorical data will be given as frequencies. The paired *t*-test will be applied to analyze continuous and categorical variables. For the safety indicators, primary endpoint, and most secondary outcomes, including blood coagulation function, routine tests (blood, urine, and excrement), biochemistry and tumor findings, the paired *t*-test will be used to detect any differences. All statistical analyses will be performed using Statistical Product and Service Solutions (Version 22.0, IBM, New York, NY, USA) by the principal biostatistician. *p* < 0.05 will be considered statistically significant.
